# Promising SARS-CoV-2 main protease inhibitor ligand-binding modes evaluated using LB-PaCS-MD/FMO

**DOI:** 10.1038/s41598-022-22703-1

**Published:** 2022-10-26

**Authors:** Kowit Hengphasatporn, Ryuhei Harada, Patcharin Wilasluck, Peerapon Deetanya, Edwin R. Sukandar, Warinthorn Chavasiri, Aphinya Suroengrit, Siwaporn Boonyasuppayakorn, Thanyada Rungrotmongkol, Kittikhun Wangkanont, Yasuteru Shigeta

**Affiliations:** 1 Center for Computational Sciences, University of Tsukuba, 1-1-1 Tennodai, Tsukuba, Ibaraki 305-8577 Japan; 2 Center of Excellence for Molecular Biology and Genomics of Shrimp, Department of Biochemistry, Faculty of Science, Chulalongkorn University, Bangkok, 10330 Thailand; 3 Center of Excellence for Molecular Crop, Department of Biochemistry, Faculty of Science, Chulalongkorn University, Bangkok, 10330 Thailand; 4 Center of Excellence in Natural Products Chemistry, Department of Chemistry, Faculty of Science, Chulalongkorn University, Bangkok, 10330 Thailand; 5 Center of Excellence in Applied Medical Virology, Department of Microbiology, Faculty of Medicine, Chulalongkorn University, Bangkok, 10330 Thailand; 6 Program in Bioinformatics and Computational Biology, Graduate School, Chulalongkorn University, Bangkok, 10330 Thailand; 7 Center of Excellence in Structural and Computational Biology, Department of Biochemistry, Faculty of Science, Chulalongkorn University, Bangkok, 10330 Thailand

**Keywords:** Proteases, Viral infection, Cheminformatics, Drug discovery

## Abstract

Parallel cascade selection molecular dynamics-based ligand binding-path sampling (LB-PaCS-MD) was combined with fragment molecular orbital (FMO) calculations to reveal the ligand path from an aqueous solution to the SARS-CoV-2 main protease (M^pro^) active site and to customise a ligand-binding pocket suitable for delivering a potent inhibitor. Rubraxanthone exhibited mixed-inhibition antiviral activity against SARS-CoV-2 M^pro^, relatively low cytotoxicity, and high cellular inhibition. However, the atomic inhibition mechanism remains ambiguous. LB-PaCS-MD/FMO is a hybrid ligand-binding evaluation method elucidating how rubraxanthone interacts with SARS-CoV-2 M^pro^. In the first step, LB-PaCS-MD, which is regarded as a flexible docking, efficiently samples a set of ligand-binding pathways. After that, a reasonable docking pose of LB-PaCS-MD is evaluated by the FMO calculation to elucidate a set of protein–ligand interactions, enabling one to know the binding affinity of a specified ligand with respect to a target protein. A possible conformation was proposed for rubraxanthone binding to the SARS-CoV-2 M^pro^ active site, and allosteric inhibition was elucidated by combining blind docking with *k*-means clustering. The interaction profile, key binding residues, and considerable interaction were elucidated for rubraxanthone binding to both M^pro^ sites. Integrated LB-PaCS-MD/FMO provided a more reasonable complex structure for ligand binding at the SARS-CoV-2 M^pro^ active site, which is vital for discovering and designing antiviral drugs.

## Introduction

The first known case of coronavirus disease 2019 (COVID-19), initially reported as idiopathic pneumonia, was confirmed in late 2019 in Wuhan City, China^1^, and has since developed into a pandemic. COVID-19 is caused by infection with the severe acute respiratory syndrome coronavirus 2 (SARS-CoV-2), which is transmitted by inhaling virus-containing droplets or contacting contaminated fluid and subsequently introducing the virus into the respiratory tract, where the virus infects the mucosa^2^. COVID-19 symptoms appear within a few days after infection. Most young and otherwise healthy people experience mild symptoms and usually completely recover within a week without taking any anti-viral agents, whereas frail elderly people with co-morbidities may experience severe symptoms and may require advanced medications and hospitalisation. The Centers for Disease Control and Prevention (CDC) has reported that the mortality rate is 65-fold higher for those aged 65–74 years than for those aged 18–29 years and is even worse in older unvaccinated groups^3^.

Numerous natural compounds inhibit the SARS-CoV-2 main protease (M^pro^)^4^, including flavonoids^5,6^, phenolics^7,8^, and xanthones^9^. Xanthones such as mangostins and their derivatives are major compounds isolated from mangosteen, which is a fruit widely used in folk medicine in tropical countries. In addition, these compounds exhibit anti-diabetic^10,11^, anti-cancerous^12^, anti-bacterial^13^, anti-malarial^14^, and anti-viral^15^ biological activities and inhibit NS2B-NS3, a dengue envelope virus protease^16^. Although molecular docking studies have recently shown that mangostins may exhibit anti-viral activity against the SARS-CoV-2 M^pro^ active site, no in vitro or in vivo studies have been conducted to date using these compounds.

SARS-CoV-2 polyprotein proteolysis is manipulated by the main protease (M^pro^) and the papain-like protease (PL^pro^), which play an essential role in the viral life cycle^17^. One of the most promising anti-SARS-CoV-2 drug targets is M^pro^, also known as 3CL protease. The enzyme catalytic dyad cleaves the viral polyproteins at specific conserved sites. Many anti-viral M^pro^ inhibitors, including Nirmatrelvir/Ritonavir (Paxlovid™)^18^ and Ensitrelvir (S-217622)^19^, work by interrupting the polyprotein cleavage, thereby inhibiting viral replication. Proteases are vital drug targets for several viruses including the human immunodeficiency virus (HIV) and the hepatitis C virus (HCV).

Several computational studies have targeted the SARS-CoV-2 M^pro^ crystal structure, which was elucidated within a few weeks after COVID-19 emerged^6,20–22^. The SARS-CoV-2 M^pro^ protein is widely used as a template to identify potential candidate drugs for further investigation and for the repurposing of clinically approved drugs and has been virtually screened using computational chemistry approaches. Each M^pro^ monomeric structure exhibits domains 1, 2, and 3 at residues 8–99, 100–183, and 184–303, respectively. The active site comprises catalytic dyads H41 and C145, which are used as landmarks to search for inhibitors^8^. Moreover, an allosteric binding region between domains 2 and 3 serves as a non-competitive inhibitor binding site^23,24^.

The active-site shape depends on the ligand that binds to the protein. Usually, molecular-docking-based virtual screening for potent compounds only enables ligands to search for the optimal configuration in the conformational space and then dock to the rigid protein. In flexible or induced-fit docking, on the other hand, although the protein side chain moves, the protein backbone does not^25,26^, which is problematic in molecular dynamics (MD) simulations using a ligand–protein complex. According to our previous work^6^, although docking and fragment molecular orbital (FMO) methods revealed that some ligands, baicalein, quercetin, and hesperetin, exhibited good binding interaction energies, the ligands did not remain in the binding pocket during the MD simulations because either the compounds did not inhibit the protein target or the ligands did not fit well into the binding pocket and dissociated from the target during short-timescale MD simulations. To overcome this limitation, a complex structure was constructed by customising a binding pocket suitable for each ligand prior to performing the MD simulations. As a similar ligand-binding sampling method, supervised molecular dynamics (SuMD) have been proposed and extensively applied to several drug targets^27^. Target protein–ligand binding pathways are rare because they are stochastically induced over accessible conventional MD simulation timescales. Therefore, developing several rare-event sampling methods such as parallel cascade selection MD (PaCS-MD) is highly desirable^28–30^. Moreover, PaCS-MD has previously been applied to several biological systems to identify rare events^31,32^.

## Results and discussion

**Figure 1:**
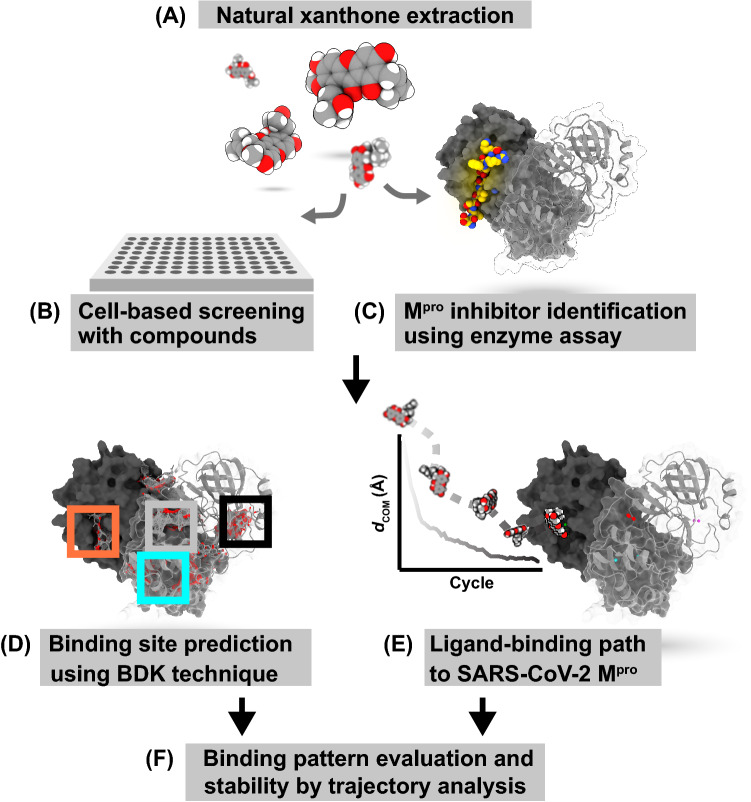
Study overview.

The study overview is shown in Fig. 1 (A). The extracted natural xanthones were used to test (B) the cytotoxicity and cellular inhibitory effect against SARS-CoV-2. These compounds were identified as inhibitors targeting M^pro^, and their inhibition mode was confirmed using a (C) protease inhibition parallel-line assay. (D) The allosteric site hits were determined by combining blind docking with *k*-means clustering (BDK). To confirm the allosteric site ligand-binding mode, MD simulations were performed for rubraxanthone complexed with M^pro^ and the M^pro^ substrate bound to the active site. We used a PaCS-MD extension called (E) ‘ligand-binding PaCS-MD’ (LB-PaCS-MD)^33^ to efficiently sample the ligand-binding pathways of the target protein (SARS-CoV-2 M^pro^) to the active site and subsequently used the FMO–RIMP2/PCM calculation to estimate the ligand/M^pro^ complex binding interaction energy. (F) The binding free-energy calculation was then used to evaluate the ligand-binding stability from a clustered MD trajectory. The compound binding pattern may be useful for designing and developing drugs.

### Experimental study

#### Xanthone derivatives as potent inhibitors of SARS-CoV-2

**Figure 2:**
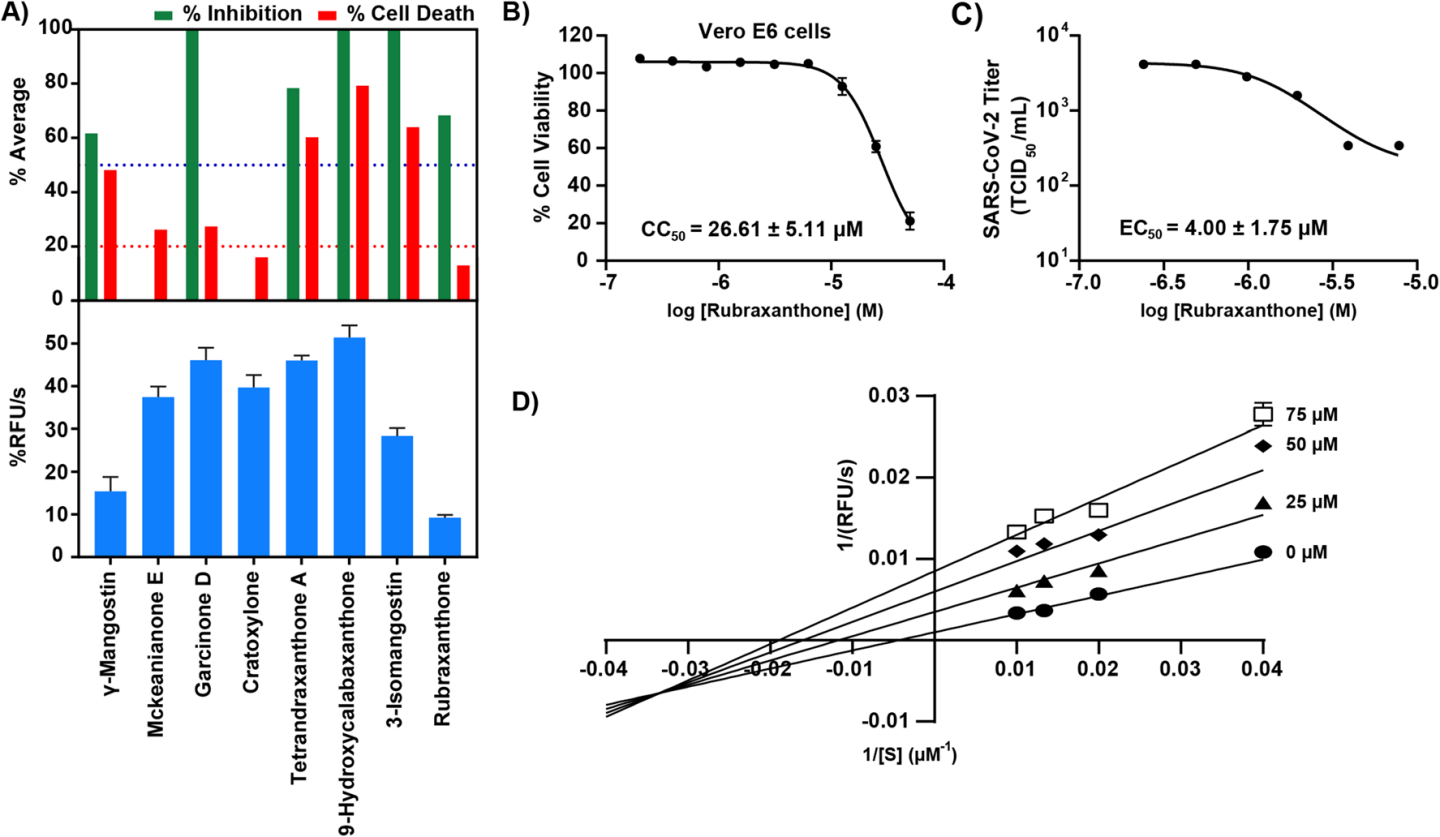
(**A**) Cell-based and SARS-CoV-2 M^pro^ inhibition screening; (**B**,**C**) effect of rubraxanthone on cell viability, as determined using MTS assay; and (**D**) Lineweaver–Burk plot of rubraxanthone-induced SARS-CoV-2 M^pro^ inhibition.

The in vitro cell-based experiments initially involved the screening of *γ*-mangostin, mckeanianone E, garcinone D, cratoxylone, tetrandraxanthone A, 9-hydroxycalabaxanthone, 3-isomangostin, and rubraxanthone at a final concentration of 10 µM in SARS-CoV-2-infected Vero E6 cells. Mckeanianone E and cratoxylone did not exhibit any viral inhibition. Garcinone D, 9-hydroxycalabaxanthone, and 3-isomangostin, exhibited both strong viral inhibition (> 99%) and high cytotoxicity (cell death > 20%) and therefore were inappropriate for further investigation. Remarkably, rubraxanthone exhibited both relatively high viral inhibition (68.30%) and low cytotoxicity (12.95%), as shown in Fig. 2A. Therefore, the 50% cytotoxic (CC_50_) and effective concentrations (EC_50_) (Fig. 2B,C, respectively), were further examined to determine the rubraxanthone efficacy, and the results are presented in Table 1.

**Table 1:** Rubraxanthone efficacy, as determined using cell-based assays. Results are presented as mean ± standard error of the mean (SEM) for three biologically independent experiments.

	EC_50_ (µM)	CC_50_ (µM)	
	Vero E6	Vero E6	Calu-3
Rubraxanthone	4.00 ± 1.75	26.61 ± 2.95	> 50

To verify that the inhibitors targeted SARS-CoV-2 M^pro^, a protease inhibition assay was applied to initially screen for SARS-CoV-2 M^pro^ inhibitory activity at high inhibitor concentrations (100 µM). The rubraxanthone and *γ*-mangostin strongly inhibited the SARS-CoV-2 M^pro^ activity. In contrast to the *γ*-mangostin, the rubraxanthone is not cytotoxic. Therefore, the rubraxanthone is a potential SARS-CoV-2 main protease inhibitor candidate, and its inhibitory mechanism was further explored. The Lineweaver–Burk plot (Fig. 2D) suggested a mixed mode of inhibition with the dissociation constant between rubraxanthone and the free enzyme (Ki) of 74.6 ± 24.1 and the dissociation constant between rubraxanthone and the enzyme-peptide substrate complex (Kiʹ) of 9.82 ± 3.17 μM.

### Computational study

#### Possible rubraxanthone binding sites

The Lineweaver–Burk plot from the protease inhibition assay suggests that the rubraxanthone might inhibit SARS-CoV-2 M^pro^ through mixed binding modes. The experimental data strongly suggested that the SARS-CoV-2 M^pro^ exhibited an allosteric inhibitor binding site, which could be either remote from or in the active site but did not overlap with the substrate. Because we could not completely exclude the possibility that the rubraxanthone is a competitive inhibitor that somewhat occupies the same binding site as the substrate, we used computational methods to further investigate the possible binding modes.

**Figure 3:**
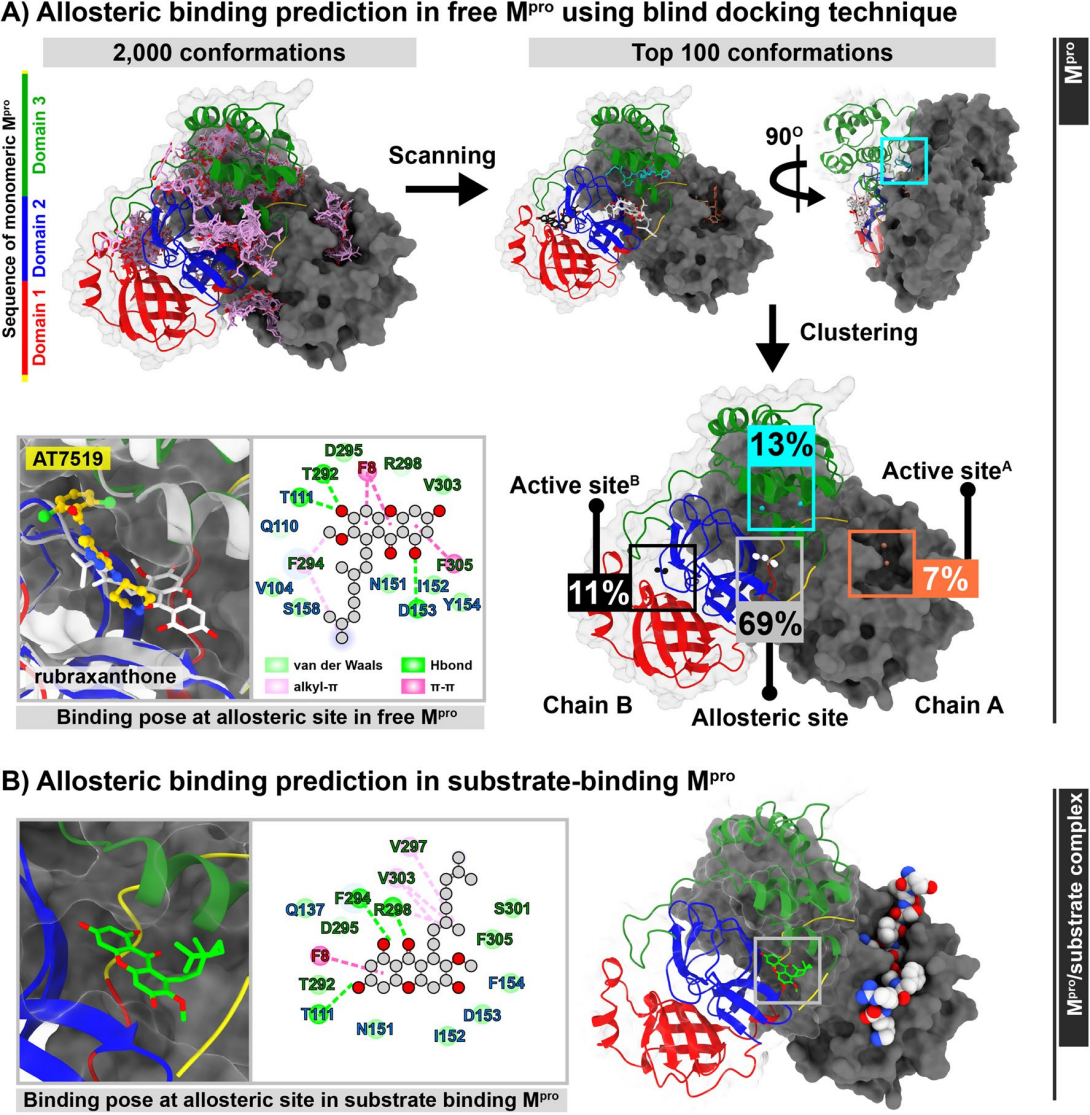
Possible rubraxanthone binding sites in SARS-CoV-2 M^pro^, as predicted using BDK. Docked poses and 2D ligand–protein interactions of rubraxanthone (**A**) and inhibitor AT7519 (PDB code: 7AGA) at free M^pro^ allosteric site and (**B**) at substrate-binding M^pro^ allosteric site.

To elucidate the inhibition mode, the possible rubraxanthone binding regions targeting the SARS-CoV-2 M^pro^ were predicted using BDK. The rubraxanthone was docked to the whole protein structure over 2000 runs using AutoDock Vina 1.2.3. The top 100 ligand conformations exhibiting the lowest binding interaction scores between − 7.55 and − 8.89 kcal/mol were grouped for determining the pre-dominant binding sites, as shown in Fig. 3A. The ligand centroids clearly showed that the rubraxanthone bound to either the allosteric site (69%) between the catalytic and dimerisation domains or the active sites (A: 7% and B: 11%)^34^ with approximately identical binding interaction energies of − 8.89, − 8.49, or − 8.47 kcal/mol, respectively, suggesting that this ligand binds to the allosteric site in the active site neighbourhood and acts as a mixed inhibitor, congruent with the enzyme assay. The allosteric site was in the groove between domains 2 and 3 at the backside of chain B, as determined for AT7519 (Fig. 3A)^35^. Interestingly, the allosteric site is not shown in the exact location on chain A owing to the different C-terminus conformations. Recently, SARS-CoV-2 M^pro^ allosteric inhibition has been studied using a computational approach, experimentally measured enzymatic activity, and an inhibition assay^36,37^.

To refine the rubraxanthone binding pose at the BDK-derived allosteric site, the focus docking method was applied to the free (A) and substrate-binding (S) M^pro^ systems using the Vina 1.2.3 software. The rubraxanthone molecule was oriented in a different conformation at the allosteric sites in both systems (Fig. 3A,B). Hydrophobic interactions, van der Waals (vdW) forces, and alkyl–π and π–π stacking had formed between the xanthone core and the residues in the C-terminated helix at the D2-3 groove and the end of the chain B C-terminus. Additionally, T111, D153, and T292 and T111, F294, and F298 hydrogen bonds stabilised the core structure in the free and substrate-binding systems, respectively. In the free system, the rubraxanthone 2,6-dimethyl-2,6-nonadiene interacts with residues V104, N151, S158, and F294 like non-competitive inhibitors in the groove^35,38^. In the peptide substrate-binding system, on the other hand, the rubraxanthone 2,6-dimethyl-2,6-nonadiene interacts with residues V297, R298, V303, S301, and F305 through hydrophobic forces. To evaluate the ligand-binding stability and strength along the simulation timescale, although the best poses of the rubraxanthone molecule binding at the enzyme and enzyme–substrate complex allosteric sites were chosen for the MD study, the possibility that the rubraxanthone is a competitive inhibitor must also be elucidated.

#### Rubraxanthone binding at allosteric site

**Figure 4:**
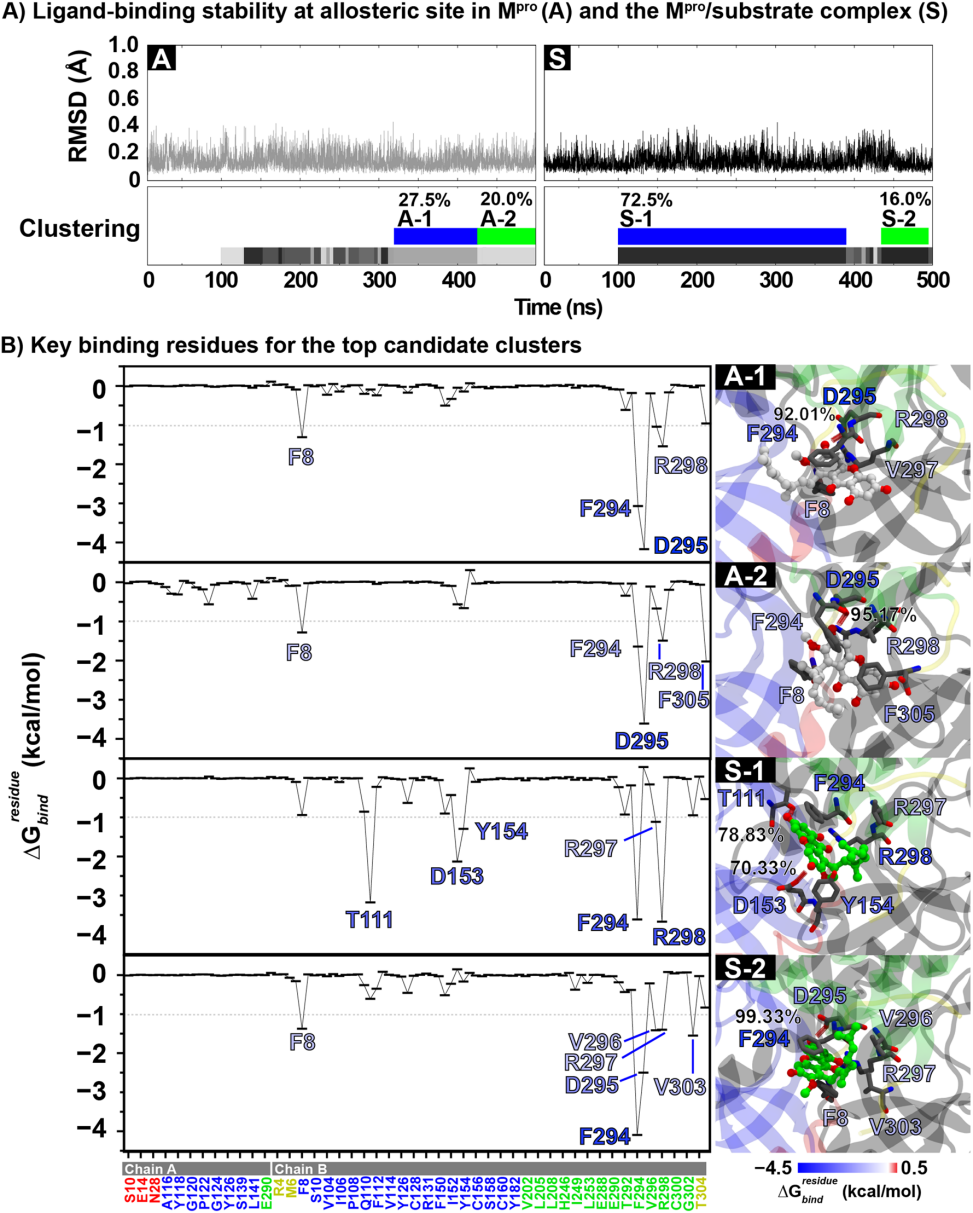
(**A**) RMSD of rubraxanthone binding at M^pro^ and M^pro^/substrate complex allosteric sites and corresponding RMSD clustering on last 400 ns trajectories. (**B**) MM/GBSA per-residue decomposition free energy ($\Delta {\text{G }}_{{{\text{bind}}}}^{{{\text{residue}}}}$) for rubraxanthone binding in top two M^pro^ and M^pro^/substrate complex clusters (A-1 and -2 and S-1 and -2, respectively). Residues responsible for ligand binding with $\Delta {\text{G }}_{{{\text{bind}}}}^{{{\text{residue}}}}$ ≤ − 1 kcal/mol and/or hydrogen bonding are depicted in right figure panel.

From the focused docking, rubraxanthone/M^pro^ complexes with and without a substrate bound at the active site were used to perform 500-ns MD simulations to evaluate the rubraxanthone binding mode at the allosteric site. According to the ligand root-mean-square deviation (RMSD), as shown in Fig. 4A, the rubraxanthone exhibited stable binding to the allosteric site of both complexes during the entire simulation period. From the RMSD clustering on the 100–500 ns trajectories, the top two clusters in the free and substrate-bound M^pro^ systems (A-1 and A-2 = 27.5 and 20.0% and S-1 and S-2 = 72.5 and 16.0%, respectively) were chosen to calculate the binding free energy using molecular mechanics with generalised Born and surface area (MM/GBSA) solvation. The data listed in Table 2 show that the substrate increased the rubraxanthone binding strength by ~ 3–5 kcal/mol. Because of the compound chemical moiety, the vdW interaction (Δ*E*_vdW_) is the major force inducing the molecular complexation with the SARS-CoV-2 M^pro^. Although rubraxanthone adopted different conformations in clusters A-1, A-2, and S-2, it exhibited an interaction profile like that of S-1 (Fig. 4B). The xanthone ring formed π–π stacking interactions with the F8, F294, and F305 and cation–π interactions with the R298 in the hydrophobic groove between domains 2 and 3. A strong hydrogen bond was detected between the D295 carboxylate group and the rubraxanthone core structure hydroxyl group in all the clusters: A-1, A-2, S-1, and S-2 = 92.01, 95.17, 78.83, and 99.33%, respectively. S-2 formed an additional hydrogen bond with D153 (70.33%). The rubraxanthone exhibited highly constant binding to the M^pro^ allosteric site responsible for the mixed inhibition mode, as suggested by the protease inhibition assay.

**Table 2:** MM/GBSA-calculated binding free energy and energy components (kcal/mol) for top two clusters of rubraxanthone binding to free enzyme and enzyme–substrate complex allosteric sites.

	M^pro^		M^pro^/substrate complex	
	A-1	A-2	S-1	S-2
Δ*E*_vdW_	− 36.37 ± 0.43	− 38.22 ± 0.47	− 39.69 ± 0.40	− 42.00 ± 0.58
Δ*E*_ele_	− 30.98 ± 1.04	− 20.71 ± 0.59	− 40.28 ± 0.77	− 27.56 ± 0.76
Δ*E*_MM_	45.65 ± 0.85	37.65 ± 0.37	48.61 ± 0.54	43.36 ± 0.76
$\Delta {\text{G}}_{{{\text{Sol}}}}^{{\text{GB/ele}}}$	− 3.70 ± 0.03	− 3.68 ± 0.03	− 3.50 ± 0.02	− 3.93 ± 0.04
$\Delta {\text{G}}_{{{\text{Sol}}}}^{{\text{GB/non - polar}}}$	− 67.35 ± 0.91	− 58.93 ± 0.55	− 79.97 ± 0.75	− 69.56 ± 1.02
$\Delta {\text{G}}_{{{\text{Sol}}}}^{{{\text{GB}}}}$	41.96 ± 0.84	33.97 ± 0.37	45.11 ± 0.53	39.43 ± 0.73
$\Delta {\text{G}}_{{{\text{total}}}}^{{\text{MM/GBSA}}}$	− 25.39 ± 0.33	− 24.97 ± 0.32	− 34.87 ± 0.35	− 30.13 ± 0.49
− *T*Δ*S*	20.03 ± 3.57	21.73 ± 1.28	26.46 ± 1.49	22.01 ± 1.16
$\Delta {\text{G}}_{{{\text{bind}}}}^{{\text{MM/GBSA}}}$	− 5.36 ± 0.79	− 3.24 ± 0.41	− 8.41 ± 0.20	− 8.13 ± 0.59

#### Rubraxanthone binding at active site

##### Ligand-binding path

**Figure 5:**
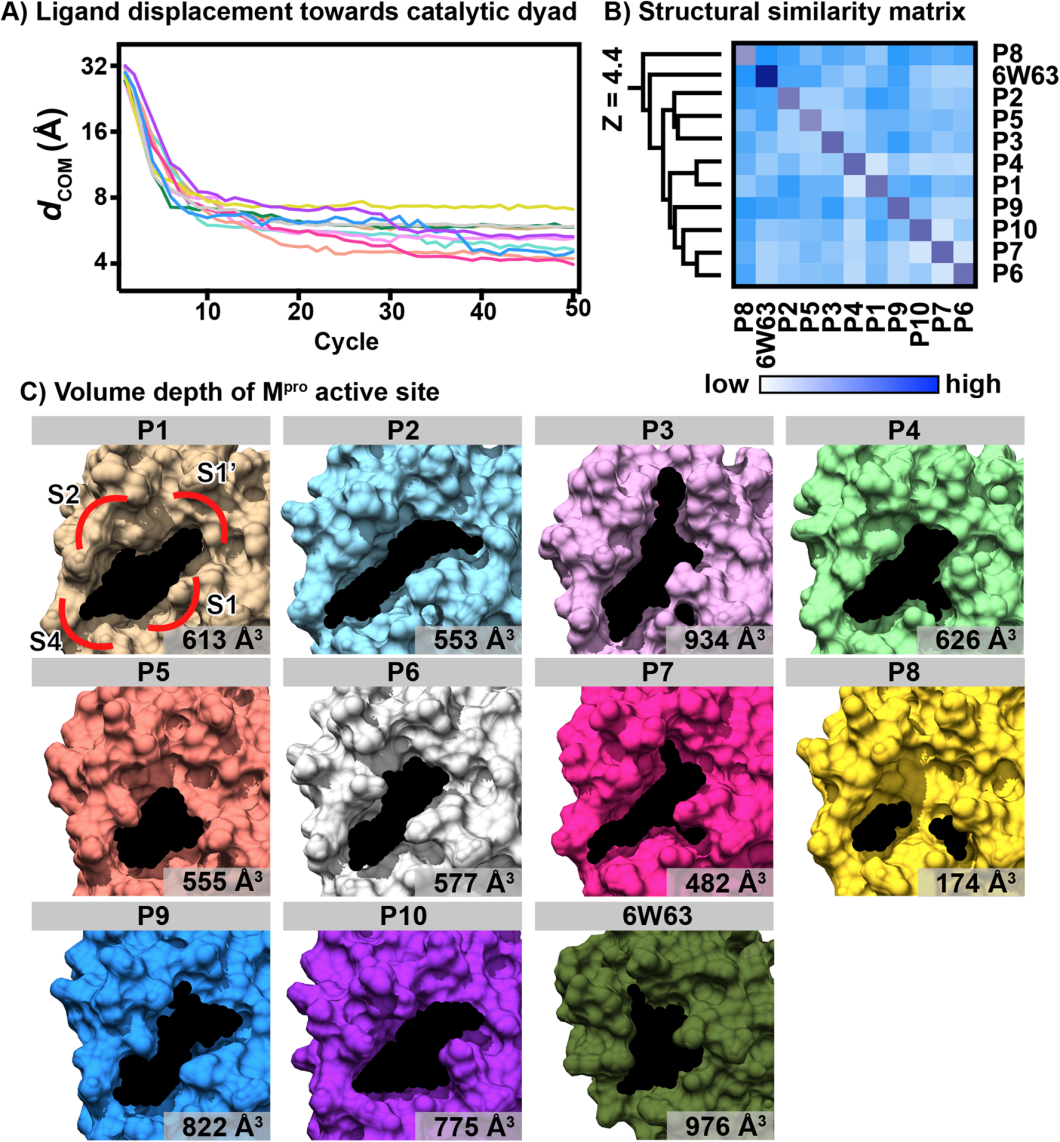
(**A**) Distance between the ligand and H41–C145 catalytic dyad centres of mass (*d*_com_) plotted for 50 LB-PaCS-MD cycles. (**B**) Pairwise structural alignment is consistent with the ligand-binding pocket at M^pro^ active site. (**C**) Corresponding volume depth compared to that of the SARS-CoV-2 M^pro^/X77 complex (6W63) crystal structure.

LB-PaCS-MD was used to determine all the possible binding paths and conformations of rubraxanthone at the M^pro^ catalytic site. This powerful technique is an enhanced sampling method used to search for a customised complex for an inhibitor using a fully structural dynamics system, which is more realistic than and superior to rigid or flexible molecular docking. Because LB-PaCS-MD can overcome the conventional docking limitations, it is suitable for customising enzymatic protein induced-fit pockets. LB-PaCS-MD directs a ligand toward a certain configuration in the binding site of a target protein by selecting ligand’s conformation with smaller *d*_COM_ values and their conformational resampling. Ten ligand-binding poses were generated from trials P1 to P10 using the same initial co-ordinates. The distance (*d*_COM_) between the ligand and target protein catalytic dyad centres of mass (COMs), as shown in Fig. 5A, suggests that in each trial system, the ligand moved towards the catalytic site at approximately the 12th cycle and then attempted to search for the optimal binding poses within the conformational space until the 50th cycle. The *d*_COM_ analysis results showed that the ligand dynamics converged after the 40th cycle in each system. As an application consideration, the selection bias in LB-PaCS-MD might stochastically make the final result towards a distorted orientation of a ligand for the binding site of a target protein. Therefore, as a careful treatment, it might be better to relax representative snapshots sampled by LB-PaCS-MD in the final cycle. To generate the most reasonable ligand-binding pose without any structural distortion, MD simulations from representative complexes sampled by LB-PaCS-MD will relax them and provide more reasonable protein–ligand orientations with fewer structural distortions at the binding site of a target protein. Therefore, the last snapshot extracted from the last cycle was selected as a representative complex for further study.

The structural similarity matrix (Fig. 5B) shows the ligand-binding pocket pairwise structural alignment for the ten complexes and the co-crystal structure of the M^pro^ active site bound with the X77 inhibitor (PDB code: 6W63) clustered by Dali Z-scores^39^. From the cladogram, the binding pocket shapes were classified as cluster 1 (P1, P4, P6, P7, P9, and P10), cluster 2 (P2, P3, and P5), and outliers (P8 and 6W63). The pocket for the rubraxanthone binding at the active site was well generated by LB-PaCS-MD, which was consistent with the ligand-binding conformation and, thus, differed from the 6W63 customised pocket (Fig. 5C). The pockets exhibited quite similar forms, aligning along the SARS-CoV-2 M^pro^ S-1ʹ and S-4 sub-pockets. The cavity volume depth was analysed using the roll algorithm implemented in the pocket cavity search application (POCASA)^40^. The solvation effect might cause this due to the LB-PaCS-MD technique that generates the pocket under the explicit water model. Among the ten trials, P3 exhibited the largest pocket (934 Å^3^), which approximated the 6W63 protein template active site size (976 Å^3^). P8 exhibited a unique structure because the S-2 pocket varied the shape of the M^pro^ binding site exhibiting the tiniest cavity (174 Å^3^). Although the order-made binding pocket technique is advantageous for generating active compound complex structures using an in-silico method, detailed molecular interactions must be considered when selecting suitable complexes for further investigation.

##### Ligand binding affinity ranking

**Figure 6:**
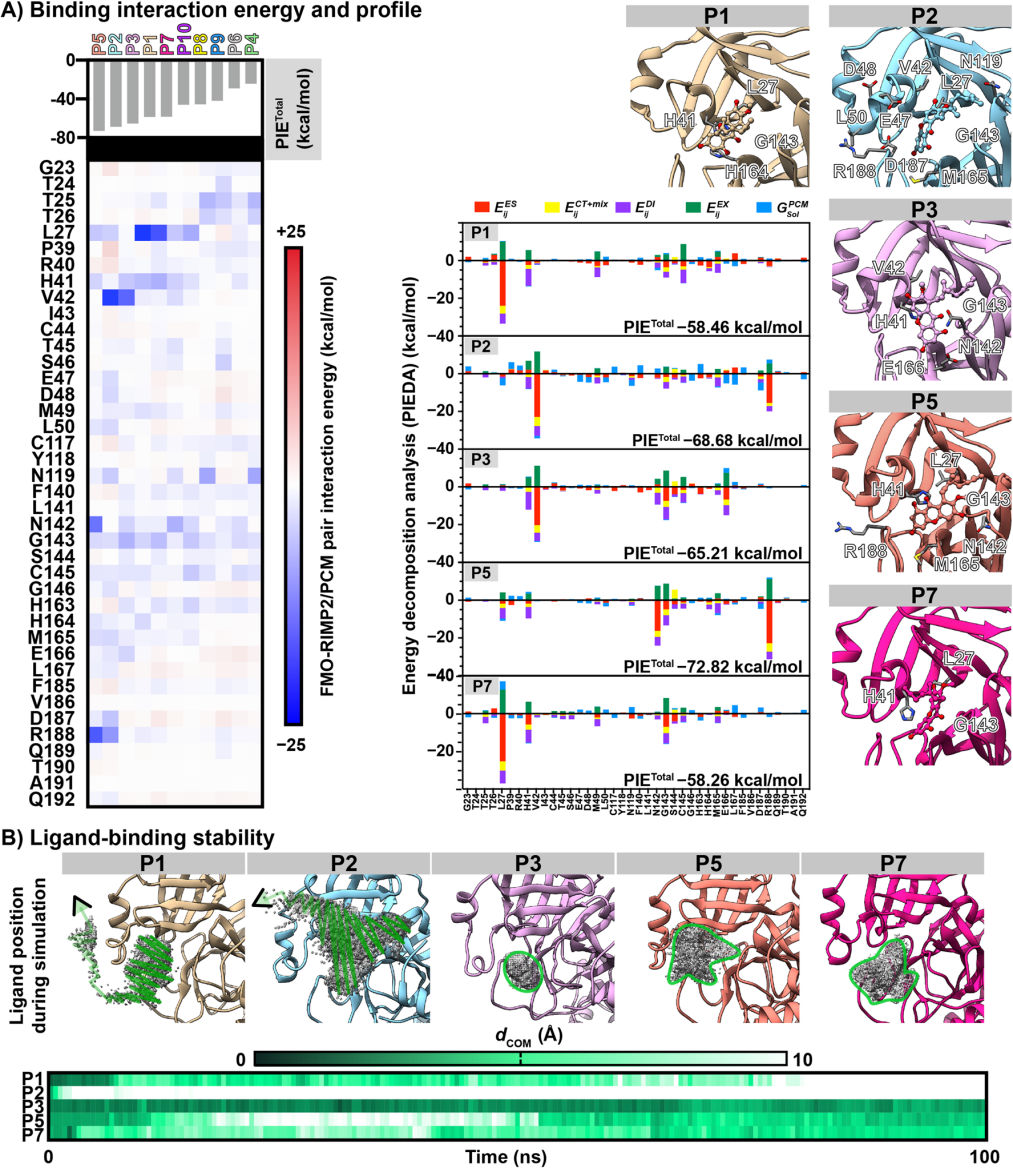
(**A**) FMO–RIMP2/PCM interaction energy profile of rubraxanthone binding at M^pro^ active site. Energy decomposition analysis (PIEDA) and total interaction energy (PIE^Total^) are presented as stacked bar graph and grid map, respectively. Key residues of top five complexes exhibiting PIE < − 1 kcal/mol are labelled. (**B**) Ligand binding distribution in active site, as determined from 100 ns MD simulation, and corresponding *d*_com_ is plotted over time.

The fragment molecular orbital (FMO) method was used to calculate the binding interaction energies of the ten rubraxanthone/M^pro^ complexes obtained using LB-PaCS-MD. The pair interaction energy (PIE) refers to the binding interaction of a ligand to each nearby residue. The profile decomposes, and the interaction between the individual residues and the ligand can be elucidated by the energy component of each binding site residue (PIEDA). According to the PIE^Total^ plot shown in Fig. 6A, the P5, P2, P3, P1, and P7 cluster 1 complexes and the other cluster 2 complexes exhibit a high binding affinity (< − 58 kcal/mol). The electrostatic ($E_{IJ}^{ES}$) and hydrophobic ($E_{IJ}^{{{\text{CT}} + {\text{mix}}}}$ and $E_{IJ}^{{{\text{DI}}}}$ in the PIEDA stacked bar graph) interactions play important roles in binding rubraxanthone at the active site. Some of the five best complexes share key binding residues such as L27 (P1, P2, and P5), H41 (P1, P3, P5, and P7), V42 (P2 and P3), N142 (P3 and P5, G143 (all systems), and R188 (P2 and P5). The P5 ligand exhibited a unique binding conformer with the lowest PIE^Total^ of − 72.82 kcal/mol.

**Figure 7:**
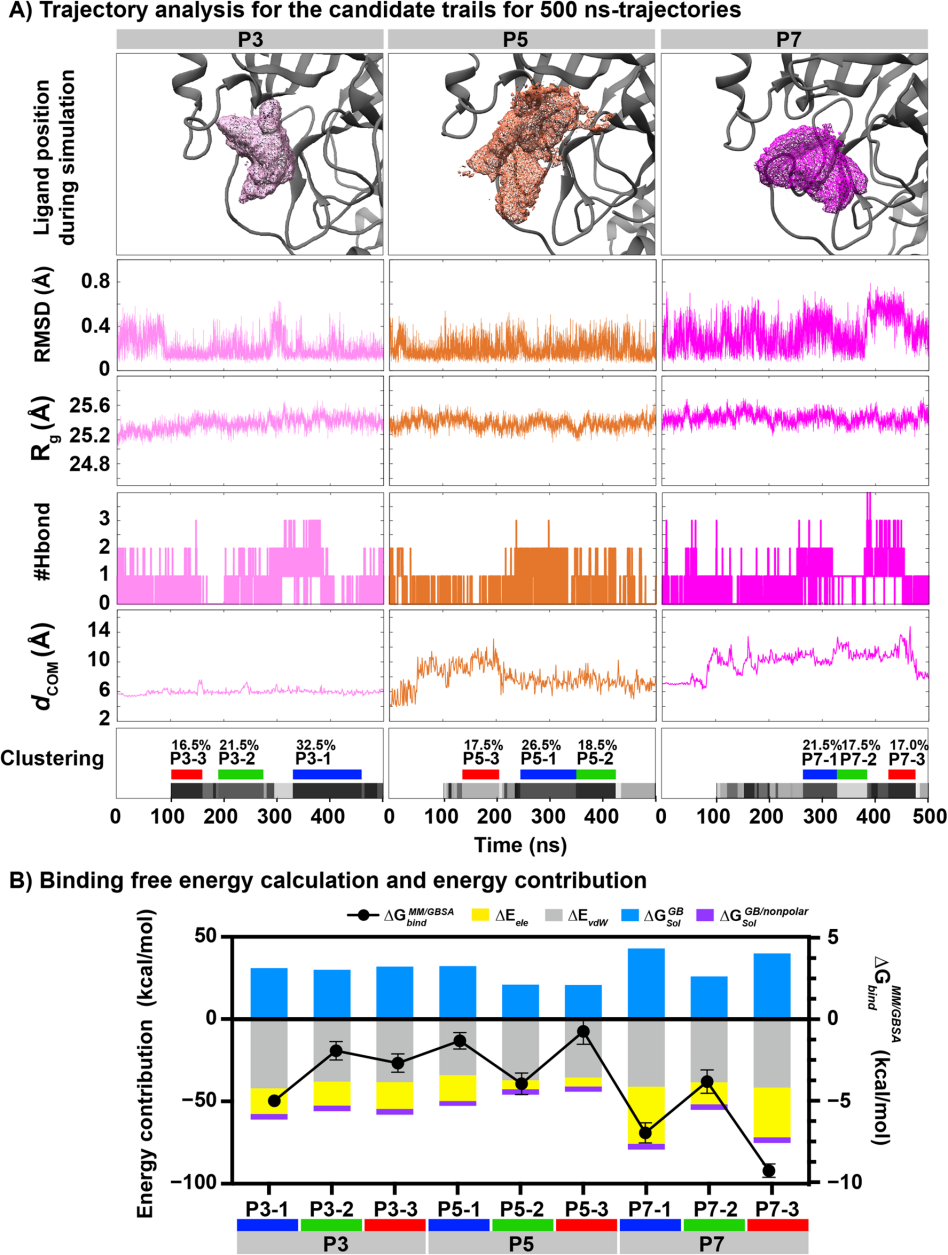
(**A**) RMSD, R_g_, hydrogen bonding, and *d*_com_ of rubraxanthone active-site binding plotted as functions of simulation time. Ligand distribution and top three clusters (P3, P5, and P7), as determined from FMO-based RMSD clustering, are shown above and below plots, respectively. (**B**) MM/GBSA-calculated binding free energy ($\Delta {\text{G}} _{{{\text{bind}}}}^{{\text{MM/GBSA}}}$) and energy components for rubraxanthone binding in P3, P5, and P7 clusters are represented by black circles and bar graphs, respectively.

The top five rubraxanthone-bound complexes were first simulated for 100 ns to investigate how the ligand occupied a conformational space at the active site. Figure 6B shows that in the P1 and P2 systems, the ligand dissociated from the protein at ~ 78 and 9 ns, respectively. In the other systems, the ligand searched for the optimal pocket position to generate stronger interactions and hold tightly in the active site. The rubraxanthone was closer to the P7 catalytic site (3.97 Å) than the P3 and P1 ones (5.22 and 5.92 Å, respectively) because the rubraxanthone had inserted the 2,6-dimethyl-2,6-nonadiene into the inner pocket. Consequently, only the P3, P5, and P7 simulations were extended to 500 ns trajectories to explore the ligand-binding pattern and strength at the M^pro^ active site. The results presented in Fig. 7A suggested that rubraxanthone can hold its position in the catalytic binding region for up to 500 ns, exhibiting RMSD values in ranges 0.07–0.63, 0.06–0.54, and 0.07–0.79 Å in the P3, P5, and P7 systems, respectively. The R_g_ of protein Cα atoms was examined to indicate the compactness of protein structure. The average Rg values of N3, N5, and N7 were ~ 25.4 Å, suggesting the tight compactness of the protein structure during 500 ns-simulation. Although the ligand moved slightly outwards from the P5 and P7 catalytic dyads by considering the *d*_com_ value (5.84 ± 1.57 and 7.24 ± 1.39 Å, respectively), ligand–protein hydrogen bonds formed throughout almost the entire 500 ns trajectories.

The ligand conformations extracted from the last 400 ns were classified by FMO-based RMSD clustering using the coordinates of the ligand and the residues exhibiting − 1 > PIE > + 1 kcal/mol. The top three clusters in each system are represented in blue/green/red (Fig. 7A), and their corresponding population percentages in the P3, P5, and P7 systems were 32.5/21.5/16.5, 26.5/18.5/17.5, and 21.5/17.5/17.0%, respectively. The MM/GBSA-calculated binding free energy ($\Delta {\text{G}} _{{{\text{bind}}}}^{{\text{MM/GBSA}}}$) and energy components of all the clusters are plotted in Fig. 7B. The top three clusters (P7-3, P7-1, and P3-1) exhibited binding affinities ($\Delta {\text{G}} _{{{\text{bind}}}}^{{\text{MM/GBSA}}}$) of − 9.13 ± 0.41, − 6.84 ± 0.61, and − 4.88 ± 0.10 kcal/mol, respectively, which were considerably higher than those of the other clusters. Although the P7-3 and P7-1 entropy contributions were similar, the rubraxanthone exhibited a higher entropy contribution in P7-1 because the ligand fluctuated in the last 400 ns. The calculated ligand-binding free energy slightly oscillated, which interfered with the catalytic site and reduced the enzyme activity.

##### Plausible active site ligand-binding mode

**Figure 8:**
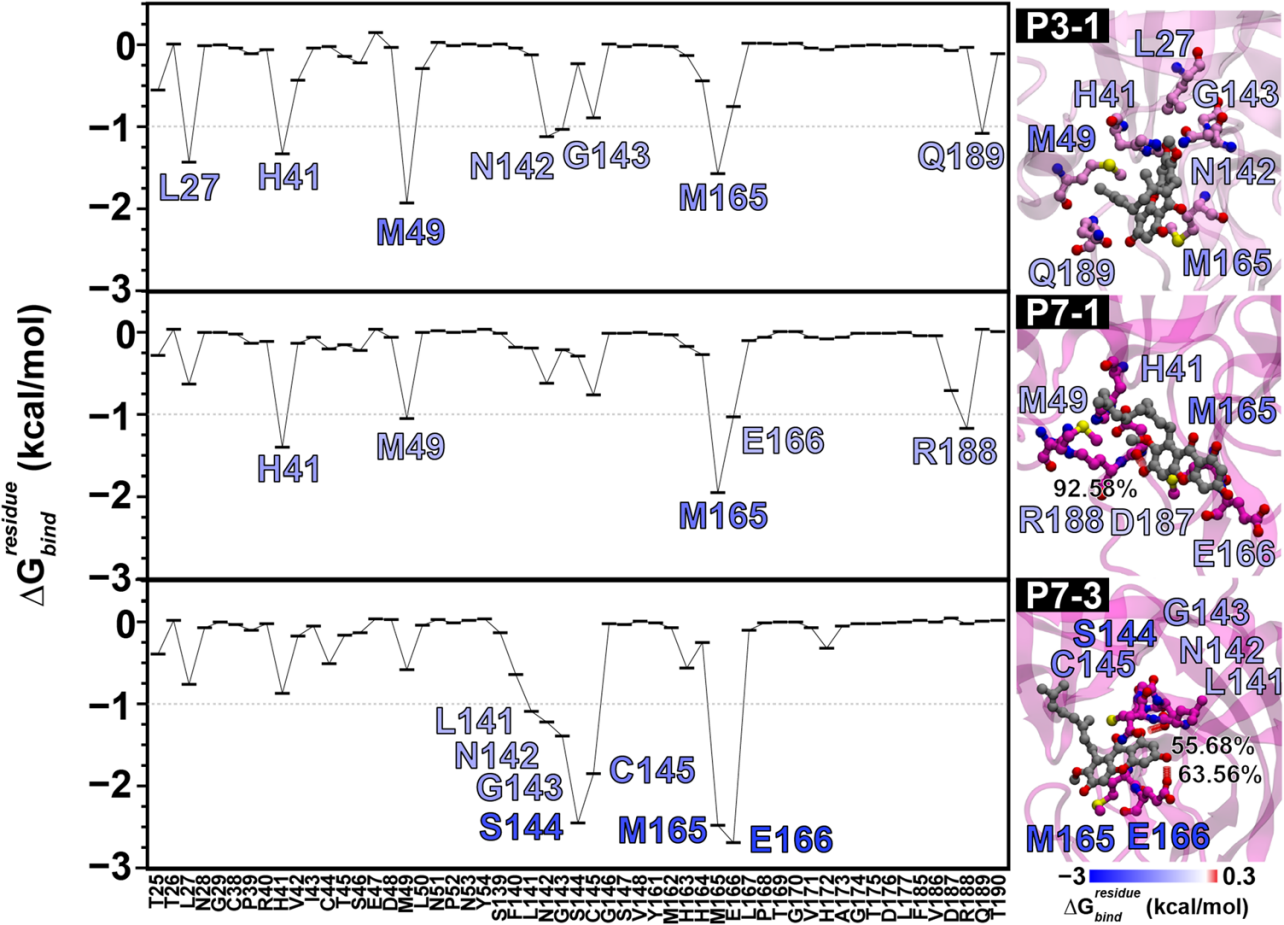
Interaction profile and key binding residues for rubraxanthone binding at M^pro^ active site in top three clusters: P3-1, P7-1, and P7-3. Residues exhibiting $\Delta {\text{G}}_{{{\text{bind}}}}^{{{\text{residues}}}}$ < − 1 kcal/mol are labelled and drawn in right figure panel.

We also decomposed the ligand/residue pairwise energetic component ($\Delta {\text{G}}_{{{\text{bind}}}}^{{{\text{residue}}}}$) of the top three clusters, which can be ranked in descending order as P7-3 > P7-1 > P3-1 (Fig. 8) based on the $\Delta {\text{G}}_{{{\text{bind}}}}^{{\text{MM/GBSA}}}$ values. The key residues contributing to rubraxanthone activity depend on the rubraxanthone binding conformation. P7-3 exhibited a unique binding pattern in which the core structure aligned in the S-1ʹ and S-4 sub-pockets, while the 2,6-dimethyl-2,6-nonadiene pointed towards the S-2 hydrophobic sub-pocket. The major contributing energy was derived from the interactions between the xanthone skeleton and oxyanion hole (residues 141–145). Hydrogen bond formation with E166 and S144 (63.56 and 55.68%) could stabilise the ligand in the active site with $\Delta {\text{G}}_{{{\text{bind}}}}^{{{\text{residue}}}}$ values of − 2.69 and − 2.45 kcal/mol, respectively. Instead, strong hydrogen bonds formed between the D187 and the xanthone core structure (92.58%) in the P7-1. Moreover, the S–π interactions between the sulphur-containing residues and the xanthone structure are essential for stabilising the ligand binding^41^. The M165 sulphur-containing residue plays a vital role in all the clusters by contributing − 2.48, − 1.95, and − 1.57 kcal/mol in P7-3, P7-1, and P3-1, respectively, and the M49 sulphur-containing residue contributes − 1.05 and − 1.93 kcal/mol in P7-1 and P3-1, respectively. The hydrophobic interactions between the 2,6-nonadiene and the residues in the S-1ʹ sub-pocket and the π–π stacking from the H41 catalytic residue to the rubraxanthone core structure contributed − 2.32, − 1.40, and − 1.33 kcal/mol in P7-3, P7-1, and P3-1, respectively, indicating that vdW interactions were the main favourable energetic contribution to the M^pro^ active-site rubraxanthone binding, as reported in previous studies^42–44^.

## Conclusion

Rubraxanthone is a promising small-molecule SARS-CoV-2 main protease inhibitor candidate that targets M^pro^ at the allosteric site and potentially at the active site. A slightly fluctuation of rubraxanthone in the binding pocket might be essential for inhibiting the M^pro^ and, thus viral replication, by interfering with the catalytic dyad and interrupting the main protease function. Rubraxanthone exhibited good M^pro^ binding profile by attaching to the allosteric binding region between domains 2 and 3 near the adjacent monomer oxyanion loop within the dimer and to the catalytic binding site through the main hydrophobic interaction contribution in both the free enzyme and enzyme–substrate complex systems. Protease inhibition assays and computational studies confirmed that rubraxanthone exhibited a mixed inhibition mechanism by interrupting both the active and allosteric sites. LB-PaCS-MD/FMO helped sample the ligand/binding conformations, which is necessary for accurately predicting complex structures and binding affinities using quantum mechanics (QM)-based calculations. The pocket shape analysis indicated that the LB-PaCS-MD could generate the suitable complex structure based on the given ligand by modifying the residue selection. A ligand interacted with the protein receptor, and the binding event could improve the program prediction accuracy. Thus, LB-PaCS-MD/FMO can be utilised to generate a customised binding conformation for potent compounds and increase the binding score accuracy, which are necessary for discovering and developing drugs.

## Materials and methods

### Experimental studies

#### Compound extraction

Garcinone D, cratoxylone, tetrandraxanthone A, 9-hydroxycalabaxanthone, 3-isomangostin, and rubraxanthone were obtained from the CH_2_Cl_2_-soluble fractions of *Garcinia cylindrocarpa* stems and *G. tetrandra* stem bark using a previously reported method^45,46^.

γ-Mangostin was purified from the pericarps of *G. mangostana*, which was collected from Purwodadi Botanical Garden, Indonesia in July 2014. The isolation process was performed as follows: The EtOAc extract (30.0 g) was subjected into silica gel column chromatography (silica gel 60, 63–200 μm; Merck, Darmstadt, Germany) with a gradient eluent of EtOAc-hexanes (0–100%) to obtain 7 fractions (A–G). Fraction D yielded from 30% EtOAc-hexanes was recrystallized using CHCl_3_:hexanes (1:1) to give γ-mangostin.

Mckeanianone E was purified from the stem bark of *G. latissima*, which was collected from North Halmahera Islands, Indonesia in March 2019. The isolation process was performed as follows: The CH_2_Cl_2_-soluble fraction (50.8 g) was loaded into silica gel column chromatography (silica gel 60, 63–200 μm, Merck, Darmstadt, Germany) with a gradient eluent of EtOAc-hexanes (0–100%) and the collected vials were grouped based on their TLC profiles (silica gel 60G F_254_, 0.25 mm; Merck, Darmstadt, Germany) to give 16 fractions (A–P). Fraction J (from 40% EtOAc-hexanes) was repeatedly separated using a Sephadex LH-20 (25–100 μm; GE Healthcare Bio-Sciences AB, Uppsala, Sweden) column to give mckeanianone E.

The ^1^H (400 MHz) and ^13^C (100 MHz) NMR spectra of the isolated compounds were recorded on a Bruker 400 AVANCE spectrometer (Bruker, Billerica, MA, USA) in CDCl_3_ and acetone-*d*_6_ (Merck, Darmstadt, Germany) and compared to the literature. The NMR spectra was shown in the Supporting Information.

#### Cell and virus cultures

Vero E6 (ATCC^®^ CRL-1587) and Calu-3 (ATCC^®^ HTB-55™) cells were purchased from ATCC (Manassas, VA, USA) and were incubated at 37 °C under 5% CO_2_ in a growth medium consisting of minimum essential medium (MEM; Gibco^®^, Langley, OK, USA) supplemented with 10% foetal bovine serum (FBS; Gibco^®^, Langley, OK), 100 I.U./mL of penicillin (Bio Basic Canada^®^, Ontario, CA), 100 µg/mL of streptomycin (Bio Basic Canada^®^, Ontario, CA), 10 mM 4-(2-hydroxyethyl)-1-piperazineethanesulphonic acid (HEPES; Sigma–Aldrich^®^, St. Louis, MO, USA), non-essential amino acid (NEAA; Gibco^®^, Langley, OK, USA), and sodium pyruvate (Gibco^®^, Langley, OK, USA).

SARS-CoV-2 Delta strain AY.85 (accession number ON381169) was isolated and propagated at 37 °C under 5% CO_2_ in Vero E6 cells in MEM supplemented with 1% FBS, 100 I.U./mL of penicillin, 100 µg/mL of streptomycin, 10 mM HEPES, NEAA, and sodium pyruvate, or maintenance media. Virus titres were measured as TCID_50_/mL in confluent cells in 96-well cell culture plates. All the experiments involving live SARS-CoV-2 were performed at a certified biosafety level 3 facility at the Research Affair Medical Research Center (MRC), Faculty of Medicine, Chulalongkorn University in Bangkok, Thailand. The study was conducted in accordance with the guidelines of the Chulalongkorn University Institutional Biosafety Committee (CU-IBC 3/64). The Institutional Review Board of the Faculty of Medicine at Chulalongkorn University approved the protocol for the use of a leftover specimen (COE 017/2021, IRB No. 297/64).

#### Primary screening and efficacy study

Vero E6 cells were seeded at 8 × 10^4^ cells per well in a 24-well plate containing a growth medium and were incubated overnight at 37 °C under 5% CO_2_. The cells were infected with SARS-CoV-2 at a multiplicity of infection (M.O.I.) of 0.1/h. The infected cells were washed with phosphate-buffered saline (PBS) and incubated with 1 mL of a maintenance medium. The compounds were prepared at the indicated concentrations in 0.1% dimethyl sulphoxide (DMSO) in the maintenance medium during and after infection. The infected cells were incubated at 37 °C for 72 h under 5% CO_2_ in a humidified chamber. The supernatants were collected for analysing the Vero E6 cell viral infectivity in TCID_50_/mL. The TCID_50_ was measured using a TCID_50_ calculator (v2.1: 20-01-2017_MB). In the primary screening, a positive control represented the TCID_50_/mL value of non-treated SARS-CoV-2-infected cells. The potential protease inhibitors were identified by the compound ability to reduce at least 90% the control TCID_50_/mL. In the effective concentration (EC_50_) study, various compound concentrations were introduced to the SARS-CoV-2-infected cells at an M.O.I. of 0.1. The viral infectivity was analysed based on the TCID50/mL of the supernatants incubated for 72 h under each condition. The data were plotted, and the EC_50_ values were calculated using non-linear regression analysis and GraphPad Prism software (La Jolla, CA, USA).

#### Cytotoxicity study

The active compound cytotoxicity was tested using the Vero E6 and Calu-3 cell lines. Each cell line was seeded at 1 × 10^4^ cells per well in 96-well plates and incubated overnight. The compounds were added at the indicated concentrations to a final DMSO concentration of 0.1%. The cells were incubated for 72 h and then the cell viability was analysed using the CellTiter 96^®^ AQueous One Solution Cell Proliferation Assay kit (Promega, Madison, WI, USA) according to the manufacturer’s protocol. The plate was analysed at *A*_450_ using a VICTORTM X3 microplate reader (PerkinElmer, Waltham, MA, USA). The 0.1%-DMSO-treated cells are referred to as the ‘100% viability control’. The acceptable compound range was defined as ≥ 80% cell viability in the cytotoxicity screening. In the cytotoxicity concentration (CC_50_) study, the cells were incubated with the various compounds at various concentrations for 72 h and were subsequently analysed using CellTiter 96^®^ AQueous One, as previously described. The cell CC_50_ values were calculated using non-linear regression analysis.

#### Protease inhibition assay

A native-terminated SARS-CoV-2 M^pro^ was produced using a previously reported procedure for producing a native-terminated SARS-CoV-1 M^pro^^47^. The protease activity and inhibition assays were performed as described in previous studies^6,21,48^.

### Computational details

#### System preparation

The 3D rubraxanthone structure was constructed using Gaussview 6, and the geometry was optimised using density functional theory (DFT) with the B3LYP/6-31G* basis set and Gaussian 16^49^. Ligand partial charges and parameters were obtained using the restrained electrostatic potential (RESP) method, the general AMBER force field 2 (GAFF2)^50^, and the AmberTools21 package antechamber module^51^.

The dimeric main protease (M^pro^) was prepared using the SARS-CoV-2 M^pro^/X-77 complex co-crystal structure (PDB code: 6W63^43^) by removing the water molecules and ligands. All the ionisable residue protonation states were assigned using the PDB2PQR web server^52^. The topology and coordination files of this protein were generated using the tLeap module and the ff19SB force fields^53^. For the substrate-binding M^pro^, the peptide substrate (TSAVLQSGFRK) coordinates were extracted from the SARS-CoV-2 complex with PDB code 4ZUH and were superimposed on the prepared M^pro^ 3D structure. Then, the complex was minimised using the protein backbone restraints for the free and substrate-binding M^pro^ systems and the AMBER20 SANDER program to relax the system^51^.

#### Rubraxanthone binding-site prediction

The possible rubraxanthone binding sites with the SARS-CoV-2 M^pro^ dimer were determined using BDK and an in-house Python script^34^. The protein and optimised rubraxanthone structures in the PDB file were converted to PDBQT file format using the ADFR package program^54^. AutoDock Vina 1.2.3^55^ was used to dock the rubraxanthone to the entire dimeric M^pro^ in a 90 × 90 × 90-Å box over 2000 iterations. The *k*-means clustering algorithm was then applied to the top 100 docking results to evaluate the potential rubraxanthone allosteric binding regions. The blind docking results were subsequently refined using focused docking. The rubraxanthone docked at the most probable free and substrate-binding M^pro^ allosteric sites in a 20 × 20 × 20-Å box.

#### LB-PaCS-MD/FMO

LB-PaCS-MD was used to construct the complex structure for rubraxanthone bound to the SARS-CoV-2 M^pro^ active site. The original PaCS-MD method efficiently sampled the transition pathways from a certain reactant to a product when a set of end-point structures (e.g., a specified reactant and product) was preliminarily known. PaCS-MD repeats short-timescale MD simulations from the necessary configurations (e.g., initial structures) to promote transitions from a certain product to a reactant. The RMSD is a simple measure that enables product-like configurations to be selected. In every cycle, PaCS-MD selects configurations exhibiting lower product RMSD values (e.g., RMSD_product_) and re-starts the short-timescale MD simulations. By repeating the conformational re-sampling cycles, RMSD_product_ converged to a low constant, indicating that PaCS-MD had sampled all the transition pathways from the reactant to the product when RMSD_product_ < a certain threshold.

LB-PaCS-MD was developed to extend the original PaCS-MD and efficiently sample the target protein ligand-binding pathways. Under the condition that end-point structures (a target protein with a completed isolated ligand and a ligand-binding complex) are known, LB-PaCS-MD searches ligand-binding pathways by repeating multiple MD simulations from reasonably selected initial structures, corresponding to a resampling cycle that consists of two steps. The first step is selecting reasonably initial structures to perform multiple MD simulations. Then, all MD snapshots are ranked by referring to a physical variable. The highly ranked MD snapshots are chosen as initial structures. The second step is to independently restart multiple MD simulations based on the reasonably selected initial structures. By repeating the cycle, LB-PaCS-MD always restarts essential configurations that tend to bind the binding site of a target protein, which efficiently searches ligand-binding pathways based on distributed computing. In this study, LB-PaCS-MD specifies the centre-of-mass (COM) distance (*d*_COM_) between rubraxanthone and the SARS-CoV-2 M^pro^ C145 catalytic residue. The ligand-binding pathways to the protein target were sampled using LB-PaCS-MD^56^. In every cycle, all the snapshots generated from the 100-ps MD simulations were ranked based on their *d*_COM_ values, and the top ten snapshots exhibiting the lowest *d*_COM_ values were used as the initial structures in the next 100-ps MD simulation cycle. By monitoring the *d*_COM_ convergence, LB-PaCS-MD was automatically terminated at the 50th cycle. To obtain reliable ligand-binding pathways, we performed 10 independent LB-PaCS-MD trials by changing the initial conditions. The representative complex derived from the last LB-PaCS-MD cycle was selected to calculate the pair interaction energy (PIE) and decomposition (PIEDA) using FMO and the resolution of the identity second-order Møller–Plesset perturbation theory (RI-MP2) integrated with PCM solvation^57,58^ according to the protocol detailed in our previous study^6^.

LB-PaCS-MD/FMO has the advantage in efficiently searching ligand-binding pathways and quantitatively evaluating ligand-binding poses. Especifically, LB-PaCS-MD does not impose any external bias in searching ligand-binding events. In contrast, most of the conventional enhanced sampling methods set external biases/forces to search ligand-binding, i.e., tunning an external bias is time-consuming and non-trivial. Moreover, combined with the QM-based binding free energy calculation using FMO, LB-PaCS-MD/FMO granted a quantitative evaluation for the ligand-binding poses searched by LB-PaCS-MD. In summary, LB-PaCS-MD/FMO applies to any target protein without tunning perturbations owing to the advantage.

#### Molecular dynamics simulations

The selected initial complex structures of rubraxanthone/M^pro^ active site and the allosteric site were simulated under periodic boundary conditions (PBCs) using the AMBER20 package program^51^. Briefly, the complex structures obtained from LB-PaCS-MD/FMO were prepared as described in the “System preparation” section. Each system was solvated in a periodic box by TIP3P water model with a distance of 12 Å from the protein surface. Sodium ions were counted to neutralize the system using the tLeap module implemented in the AMBER 20 package. Topology and the initial coordinates generated by tLeap were gradually minimized and structurally relaxed by harmonic potentials using the SANDER program. The system was heated to 300 K for 20 ps with the canonical ensemble (NVT). The selected systems were simulated for 100 ns to evaluate the ligand-binding stability over the MD trajectory. The stable complex simulations were extended to 500 ns. FMO-selection-based RMSD clustering was then applied to each trajectory to characterise the representative snapshots and further analyse the ligand-binding pattern and susceptibility using the MM-GBSA method^59^.

## Supplementary Information

Supplementary Figures.

## Data Availability

The 3D structure of the M^pro^ complexed with a small-molecule inhibitor (X77), PDB code: 6W63, is available from the RCSB Protein Data Bank (https://www.rcsb.org/). AutoDock VinaXB (https://github.com/sirimullalab/vinaXB) was used to construct the complex structures. The FMO calculations were computed using GAMESS software (https://www.msg.chem.iastate.edu/gamess/). The molecules were visualised and the compound structures were constructed using Chimera USFC (https://www.cgl.ucsf.edu/chimera/) and VMD 1.9.3 (https://www.ks.uiuc.edu/Risearch/vmd/), respectively, which are free for academic users. Gnuplot (http://www.gnuplot.info) and Adobe Illustration 25.4.1 (https://www.adobe.com/products/illustrator.html) were used for plotting the data and visualising the graphics. The data analysis scripts and other data are available from the authors upon request.
