# Supplementary material for: Promising SARS-CoV-2 main protease inhibitor ligand-binding modes evaluated using LB-PaCS-MD/FMO
Source: Sci Rep. 2022 Oct 26;12:17984. doi: 10.1038/s41598-022-22703-1 (PMC9606277; doi:10.1038/s41598-022-22703-1)
Supplement: Supplementary file 1 — Supplementary Figures. [file 41598_2022_22703_MOESM1_ESM.pdf]

Table of Contents

Figure S1. ^{1}H NMR spectrum of γ-mangostin in acetone-d_6 2
Figure S2. ^{13}C NMR spectrum of γ-mangostin in acetone-d_6 2
Figure S3. ^{1}H NMR spectrum of garcinone D in acetone-d_6 3
Figure S4. ^{13}C NMR spectrum of garcinone D in acetone-d_6 3
Figure S5. ^{1}H NMR spectrum of cratoxylone in acetone-d_6 4
Figure S6. ^{1}H NMR spectrum of tetrandraxanthone A in $CDCl_3$ 4
Figure S7. ^{13}C NMR spectrum of tetrandraxanthone A in $CDCl_3$ 5
Figure S8. ^{1}H NMR spectrum of 9-hydroxycalabaxanthone in $CDCl_3$ 5
Figure S9. ^{13}C NMR spectrum of 9-hydroxycalabaxanthone in $CDCl_3$ 6
Figure S10. ^{1}H NMR spectrum of 3-isomangostin in $CDCl_3$ 6
Figure S11. ^{13}C NMR spectrum of 3-isomangostin in $CDCl_3$ 7
Figure S12. ^{1}H NMR spectrum of rubraxanthone in acetone-d_6 7
Figure S13. ^{13}C NMR spectrum of rubraxanthone in acetone-d_6 8
Figure S14. ^{1}H NMR spectrum of mckeanianone E in acetone-d_6 8

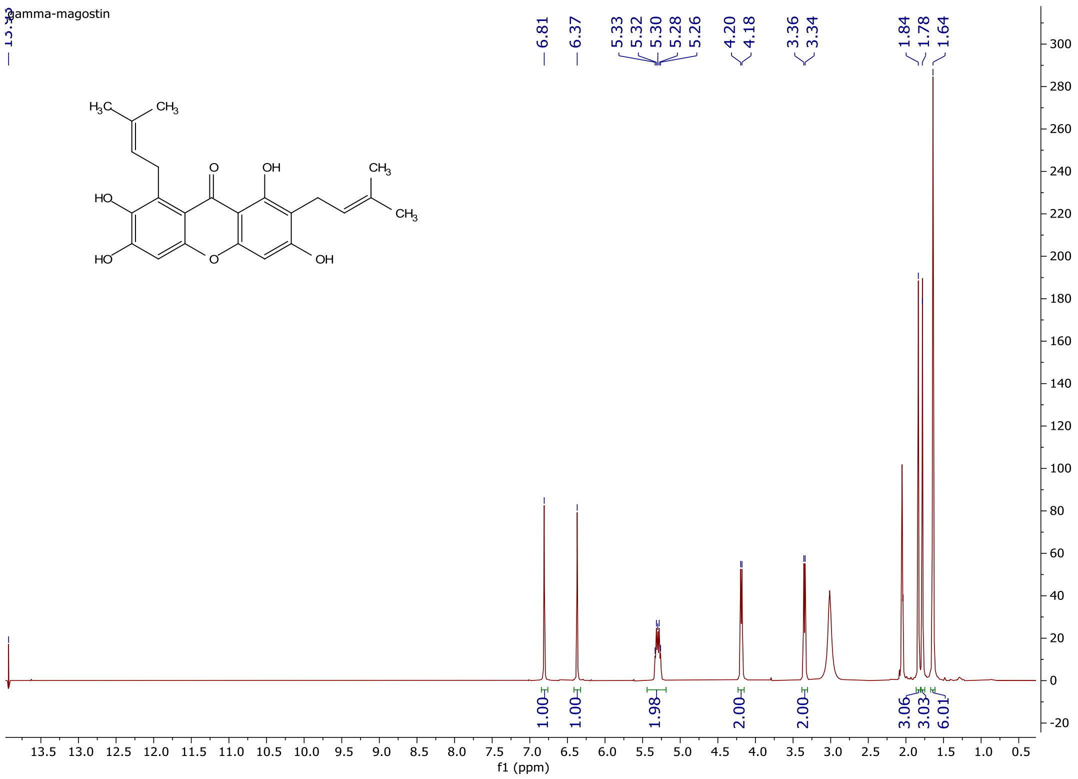

Figure S1. ^{1}H NMR spectrum of γ-mangostin in acetone-d_6

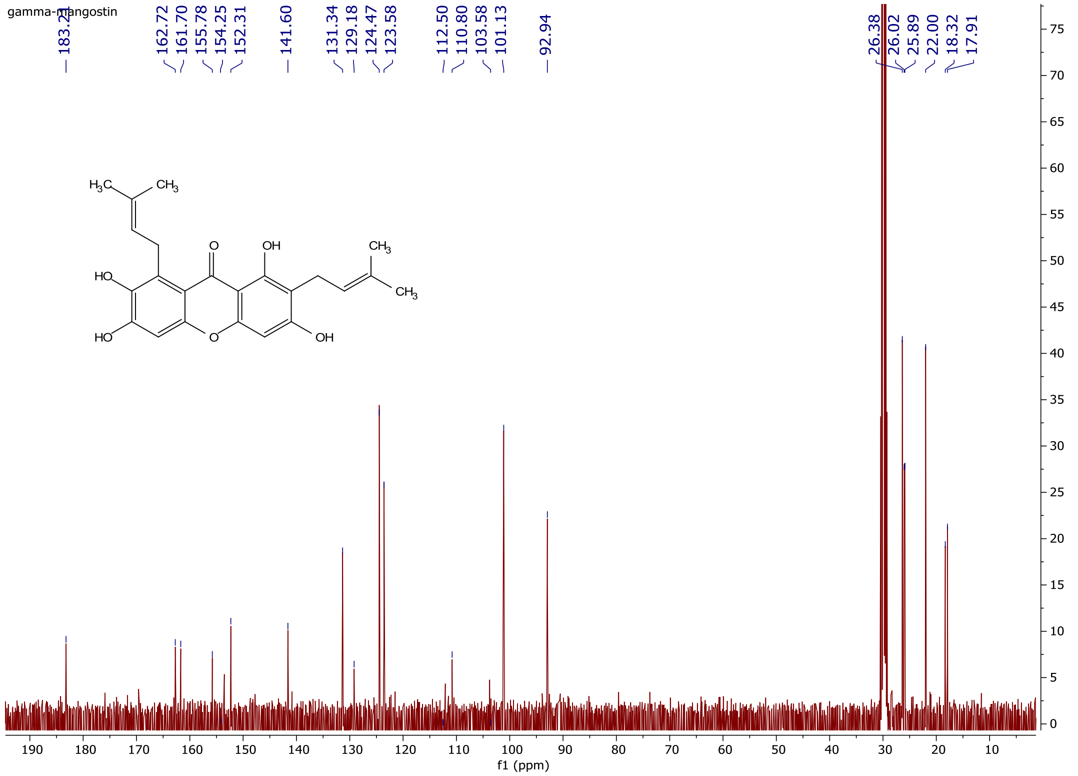

Figure S2. ^{13}C NMR spectrum of γ-mangostin in acetone-d_6

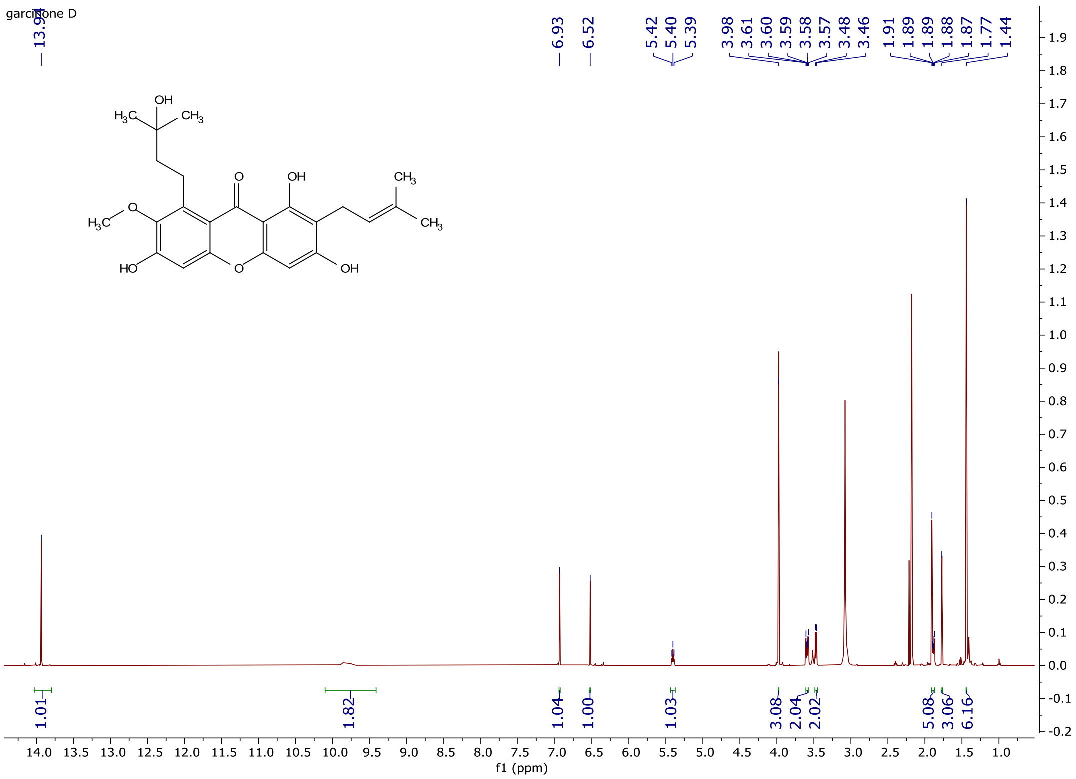

Figure S3. ^{1}H NMR spectrum of garcinone D in acetone-d_6

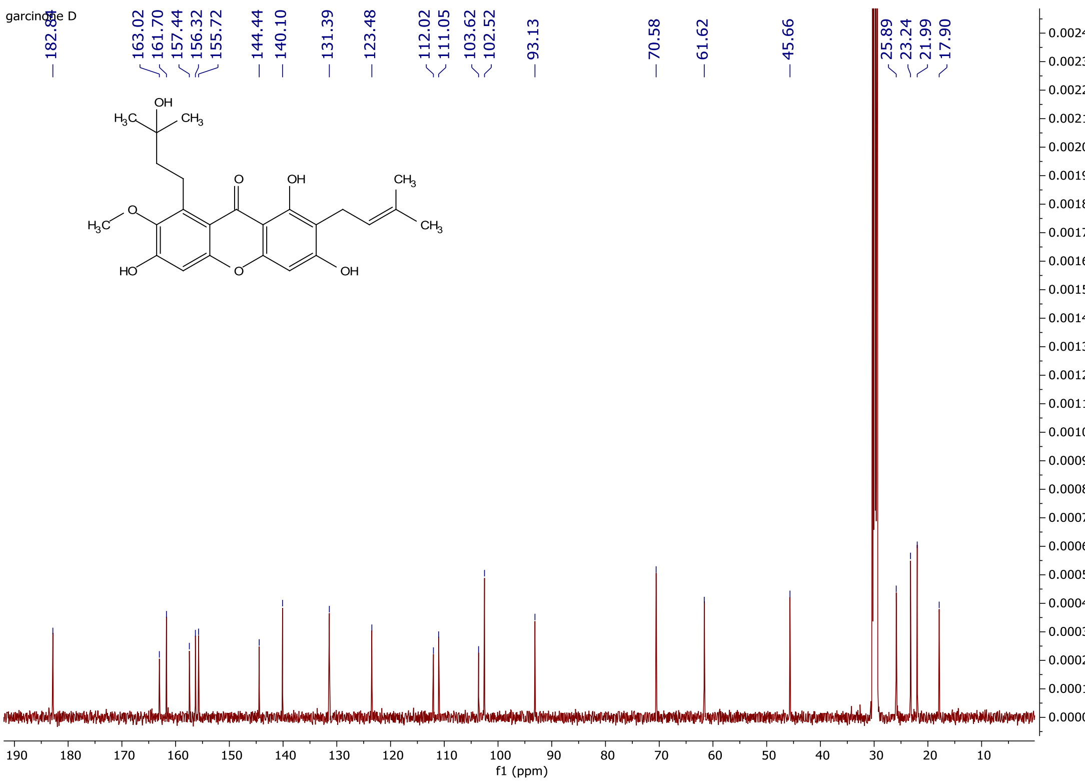

Figure S4. ^{13}C NMR spectrum of garcinone D in acetone-d_6

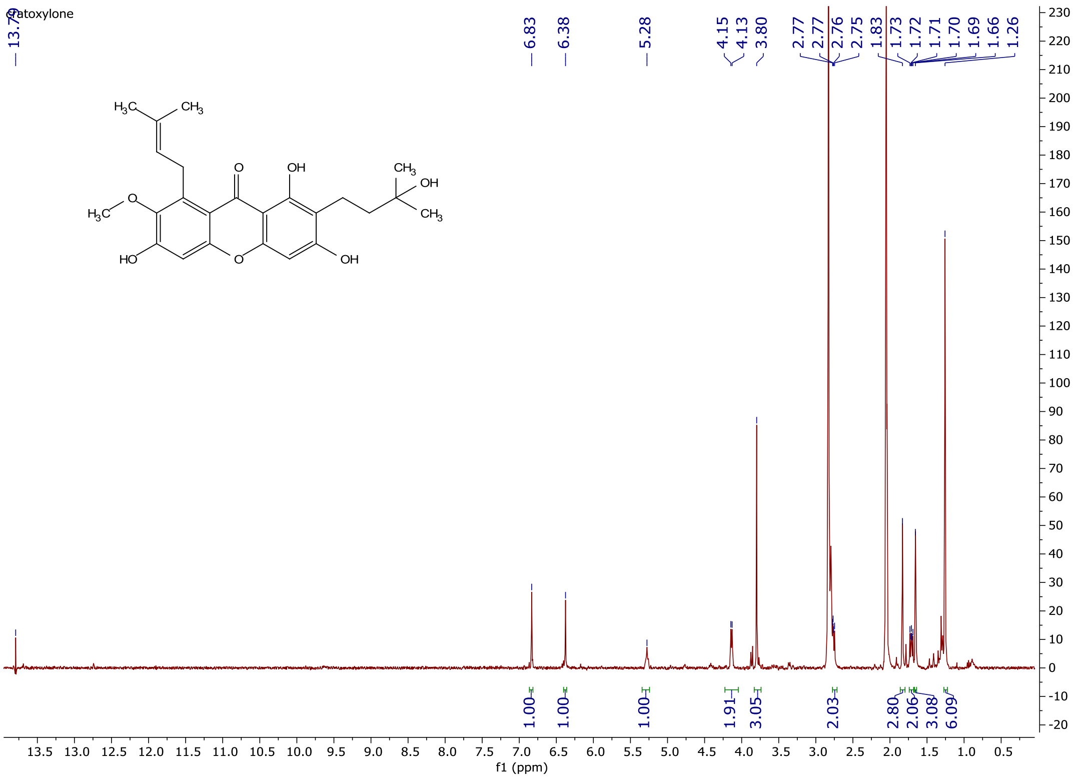

Figure S5. ^{1}H NMR spectrum of cratoxylone in acetone-d_6

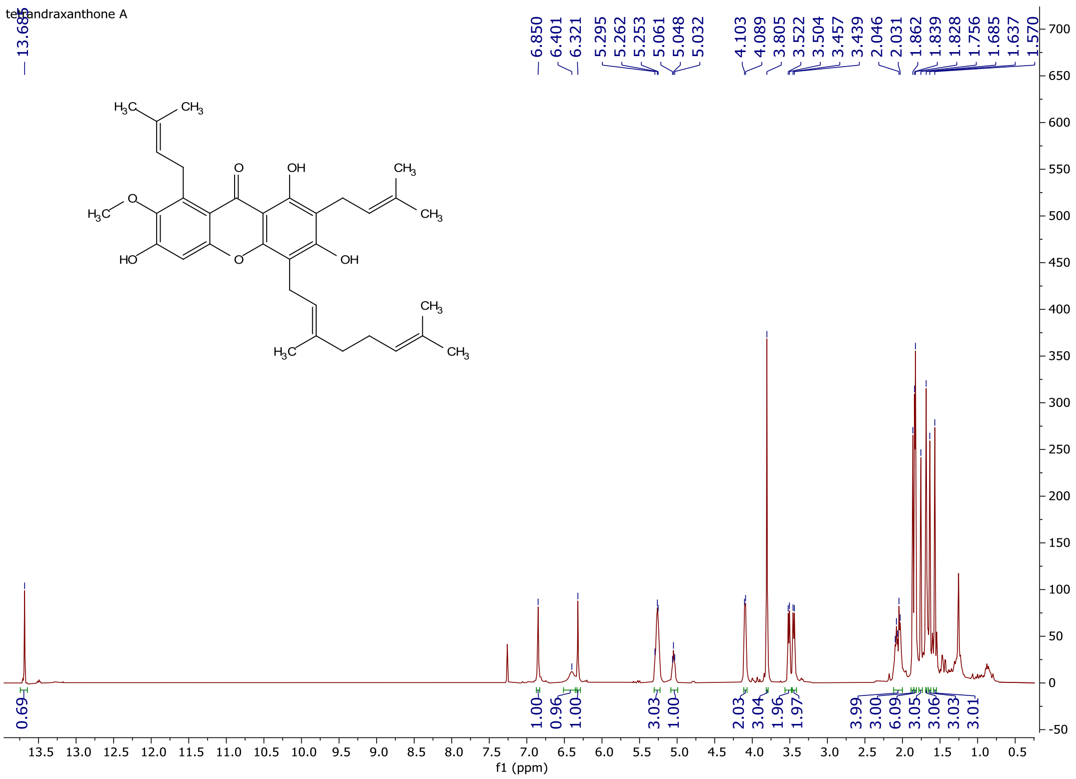

Figure S6. ^{1}H NMR spectrum of tetrandraxanthone A in $CDCl_3$

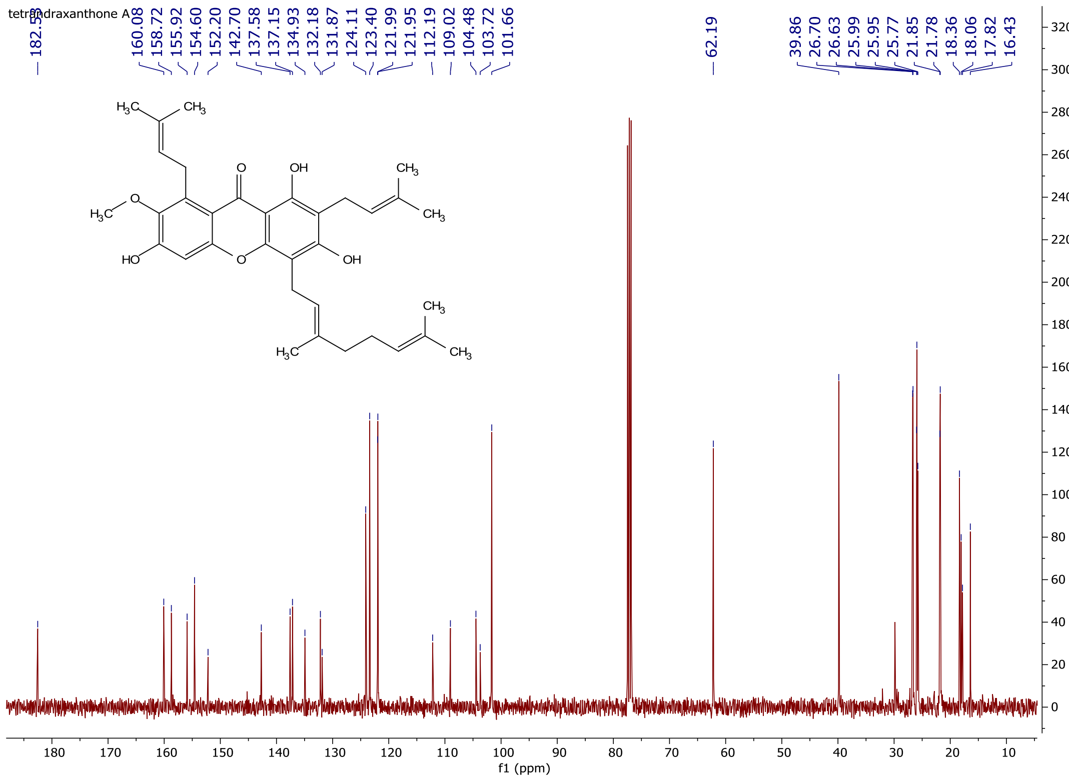

Figure S7. ^{13}C NMR spectrum of tetrandraxanthone A in $CDCl_3$

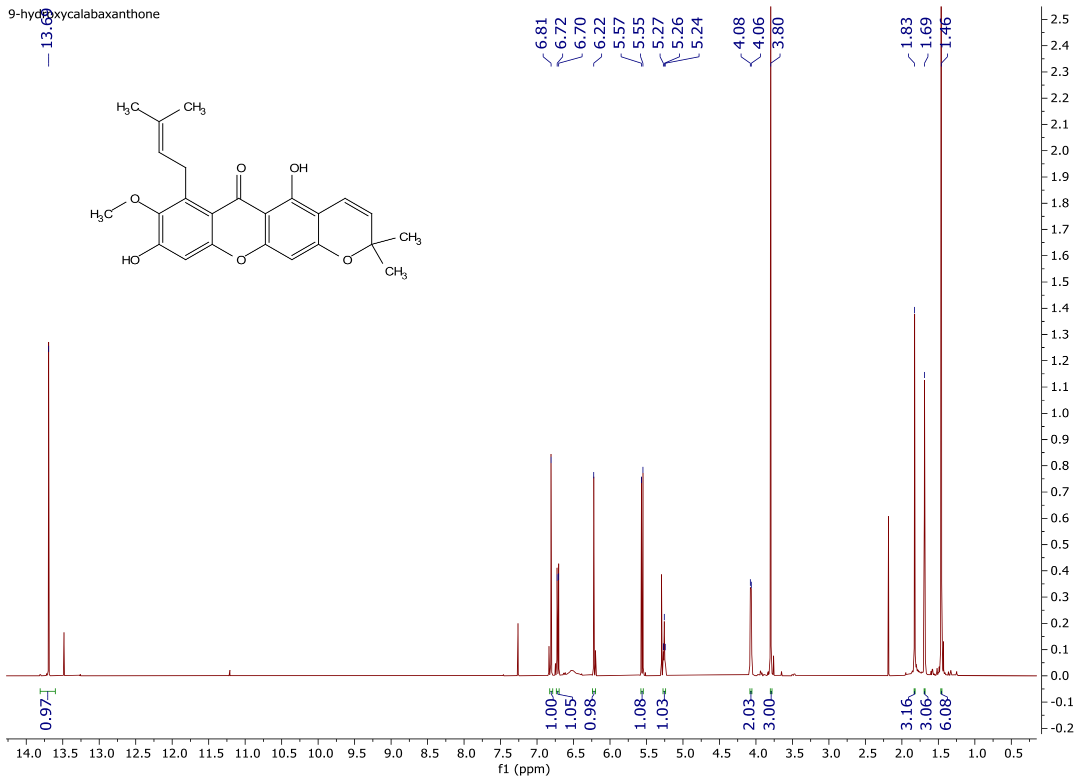

Figure S8. ^{1}H NMR spectrum of 9-hydroxycalabaxanthone in $CDCl_3$

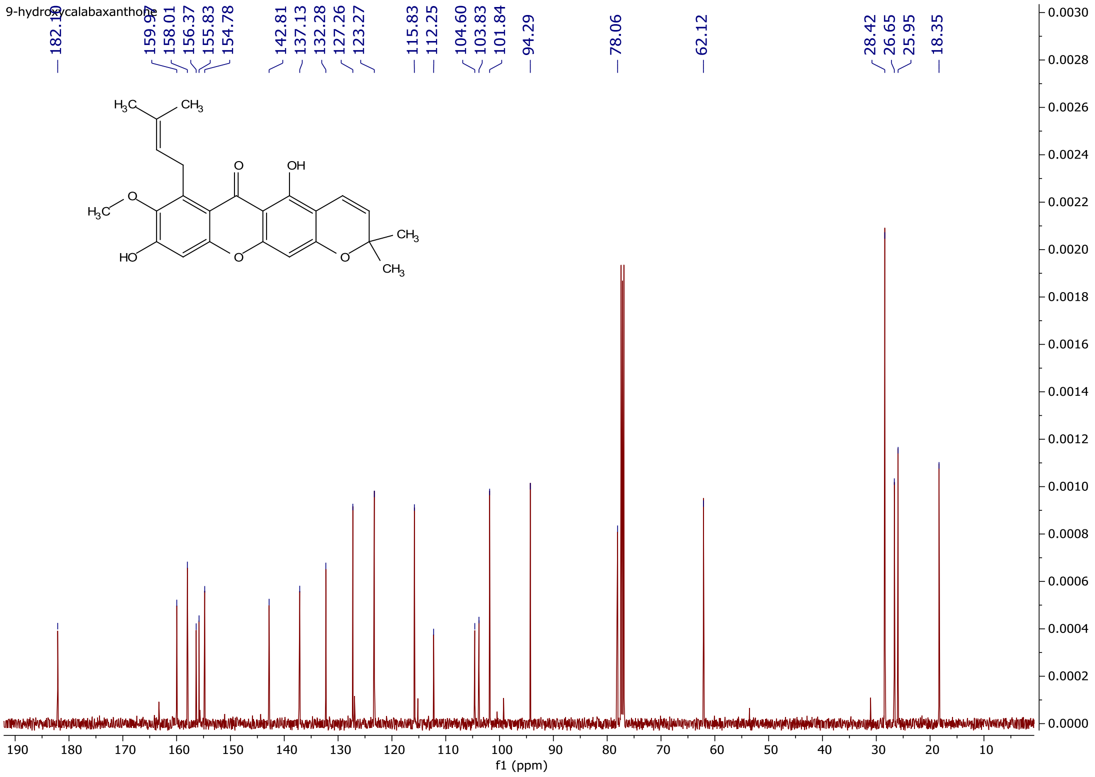

Figure S9. ^{13}C NMR spectrum of 9-hydroxycalabaxanthone in $CDCl_3$

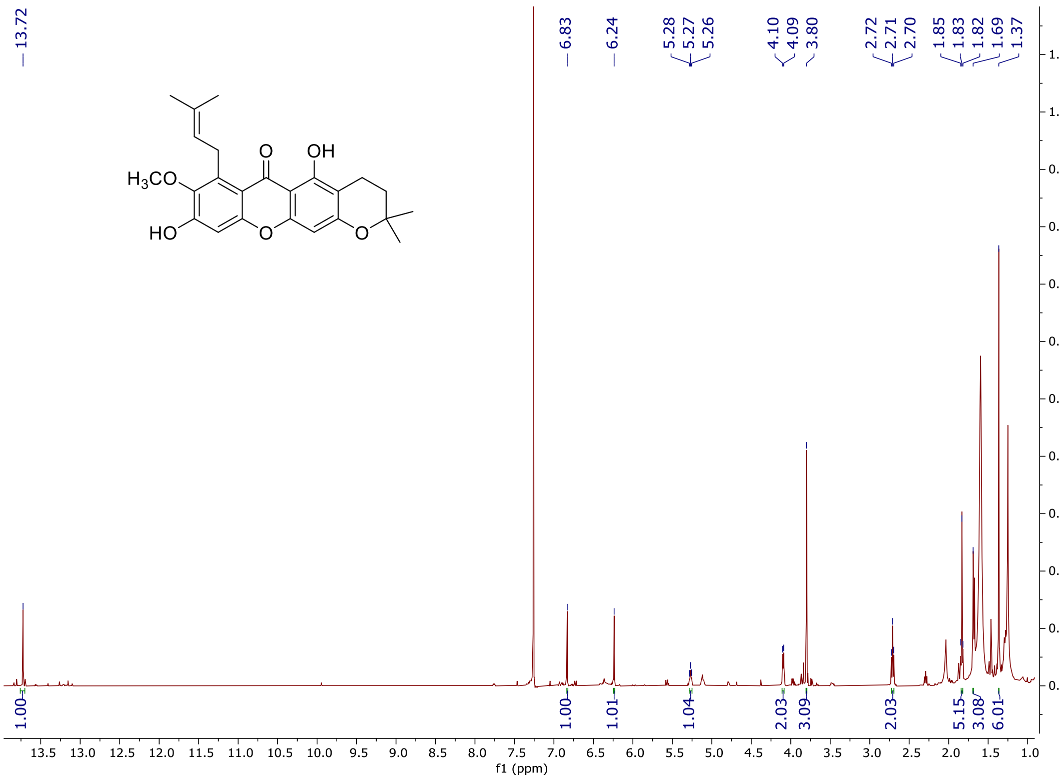

Figure S10. ^{1}H NMR spectrum of 3-isomangostin in $CDCl_3$

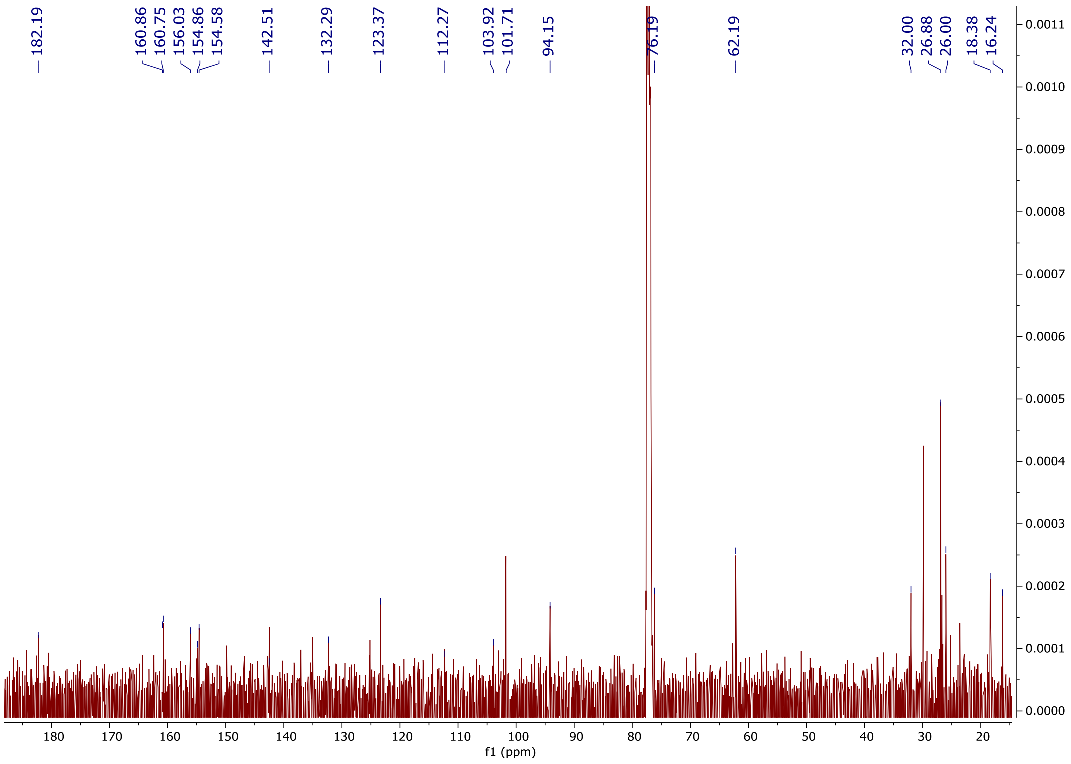

Figure S11. ^{13}C NMR spectrum of 3-isomangostin in $CDCl_3$

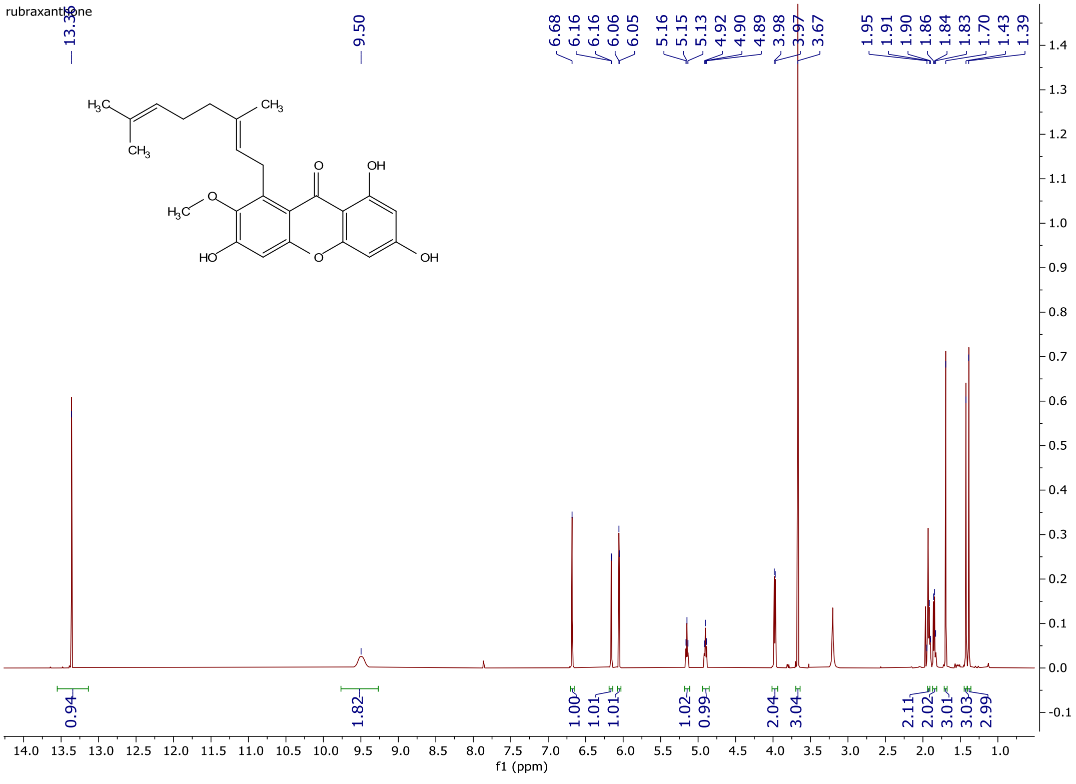

Figure S12. ^{1}H NMR spectrum of rubraxanthone in acetone-d_6

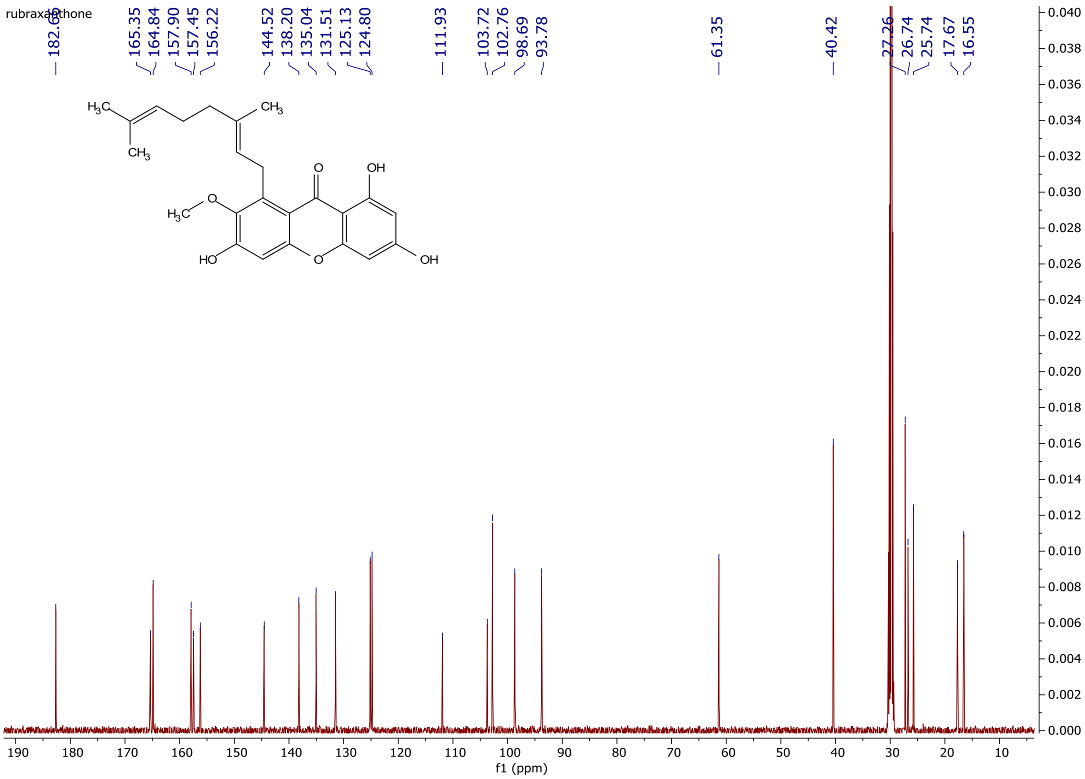

Figure S13. ^{13}C NMR spectrum of rubraxanthone in acetone-d_6

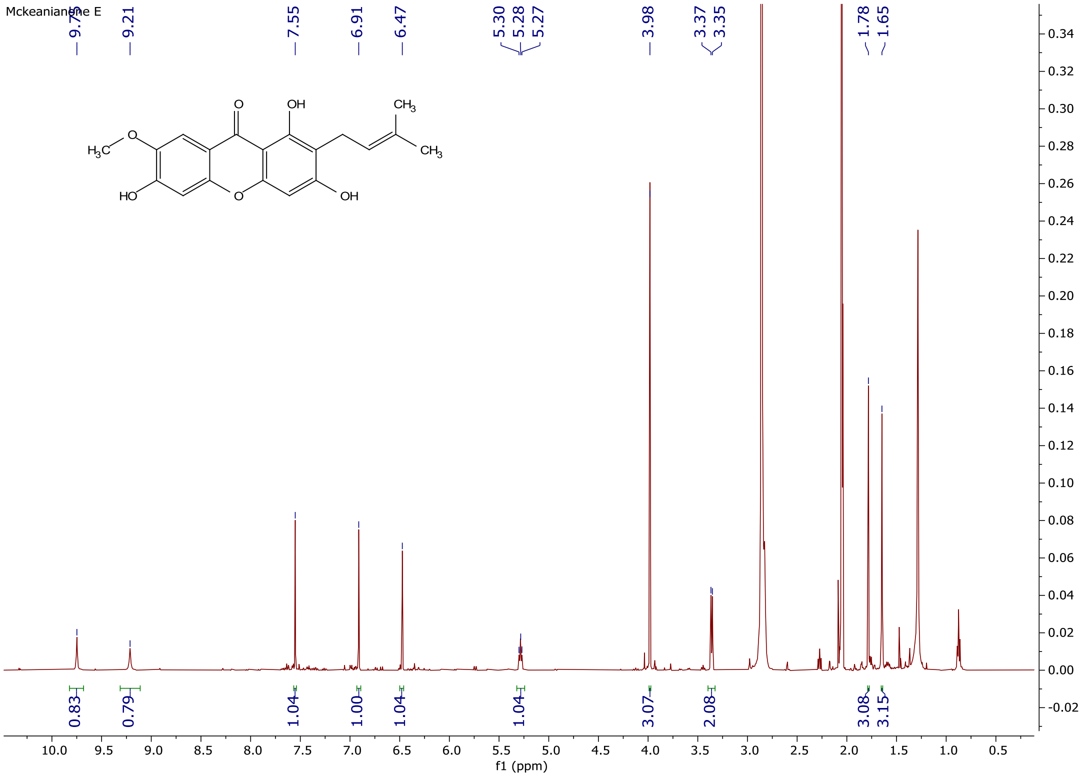

Figure S14. ^{1}H NMR spectrum of mckeanianone E in acetone-d_6
